# RNAseq shows an all-pervasive day-night rhythm in the transcriptome of the pacemaker of the heart

**DOI:** 10.1038/s41598-021-82202-7

**Published:** 2021-02-11

**Authors:** Yanwen Wang, Cali Anderson, Halina Dobrzynski, George Hart, Alicia D’Souza, Mark R. Boyett

**Affiliations:** 1 Division of Cardiovascular Sciences, University of Manchester, Manchester, UK; 2 Department of Biomedical Sciences, University of Copenhagen, Blegdamsvej 3, 2200 Copenhagen, Denmark

**Keywords:** Physiology, Cardiology

## Abstract

Physiological systems vary in a day-night manner anticipating increased demand at a particular time. Heart is no exception. Cardiac output is primarily determined by heart rate and unsurprisingly this varies in a day-night manner and is higher during the day in the human (anticipating increased day-time demand). Although this is attributed to a day-night rhythm in post-translational ion channel regulation in the heart’s pacemaker, the sinus node, by the autonomic nervous system, we investigated whether there is a day-night rhythm in transcription. RNAseq revealed that ~ 44% of the sinus node transcriptome (7134 of 16,387 transcripts) has a significant day-night rhythm. The data revealed the oscillating components of an intrinsic circadian clock. Presumably this clock (or perhaps the master circadian clock in the suprachiasmatic nucleus) is responsible for the rhythm observed in the transcriptional machinery, which in turn is responsible for the rhythm observed in the transcriptome. For example, there is a rhythm in transcripts responsible for the two principal pacemaker mechanisms (membrane and Ca^2+^ clocks), transcripts responsible for receptors and signalling pathways known to control pacemaking, transcripts from genes identified by GWAS as determinants of resting heart rate, and transcripts from genes responsible for familial and acquired sick sinus syndrome.

## Introduction

Resting heart rate is associated with cardiovascular health: an elevated resting heart rate is an independent risk factor for cardiovascular mortality and morbidity even in healthy individuals^1–3^, whereas a slow heart rate can compromise cardiac output and even lead to heart failure^4–7^. Heart rate is affected by many factors such as pregnancy^8^, development^9^ and ageing^10^, physical activity, long-term physical training^11^ and disease^12^. Another factor is the time of day and night—the resting heart rate oscillates from day to night and is lower at night in the case of diurnal species such as the human^13^. Clinically, this is important because bradyarrhythmias (slow heart rhythms because of sinus bradycardia or atrioventricular block) occur at night in humans (diurnal) and during the day in rats (nocturnal)^13–15^. This is particularly evident in veteran athletes, who have nocturnal pauses between heart beats; the longest documented nocturnal pause is 15 s^14^. Based principally on heart rate variability e.g.^16^, but also in part on autonomic blockade^17^, the day-night rhythm in heart rate is attributed to high vagal tone during sleep and changes in ionic conductances (as a result of a *post-translational* regulation of the corresponding ion channels) in the pacemaker of the heart, the sinus node. However, it is possible that the day-night rhythm in heart rate is the result of *transcriptional* changes in the sinus node. Most day-night rhythms are the result of a circadian clock and, whereas there is a master circadian clock in the suprachiasmatic nucleus, there are peripheral clocks in peripheral tissues. The heart is known to have its own circadian clock, and 6–13% of the transcriptome of the mouse heart (likely to be exclusively or mainly the ventricles) has been reported to vary in a day-night manner, presumably under the control of the local clock in the heart^18–21^ or the master circadian clock in the suprachiasmatic nucleus. In ventricular muscle, a day-night rhythm in 10 ion channel transcripts has been reported^13,22^. As well as ion channel expression, a day-night rhythm has been reported in metabolism in the heart e.g.^23^.

The aim of the present study was to measure the transcriptome of the sinus node using RNAseq and determine whether there is a functional circadian clock and a day-night rhythm in pacemaker genes in the sinus node. Because RNAseq yields the whole transcriptome of a tissue, a secondary aim was to determine if other systems in the sinus node cell, especially those which may impact pacemaking, show a day-night rhythm. The data show an all-pervasive day-night rhythm in the transcriptome of the pacemaker of the heart, the sinus node—there is a day-night rhythm in pacemaker genes and in many other systems as well.

## Results

In total, 55,450 transcripts were identified in the mouse sinus node. Expression of the transcripts was measured at six time points (at 4 h intervals) over 24 h. At each time point, expression was measured in three mice. The average expression of each transcript over 24 h was calculated and it varied from 910,910 to 0 normalised reads. The average expression of 16,387 transcripts with an expression greater than 10 normalised reads is plotted in Fig. S1A in the Data Supplement. It was assumed that transcripts poorly expressed do not play a functional role in the sinus node cell, and transcripts with an average expression of less than 10 normalised reads were arbitrarily excluded from further analysis. JTK Cycle software was developed by Hughes et al.^24^ to test whether a variable shows a statistically significant day-night rhythm. The 16,387 transcripts plotted in Fig. S1A were analysed using JTK Cycle and Fig. S1B shows that of these 7134 transcripts (~ 44%) showed a significant day-night rhythm (permutation-based *P* value < 0.05). Figure S1C shows the amplitude of the day-night variation, i.e. deviation from the mean; the day-night variation from day to night (2 × amplitude) could be substantial. Figure S1 demonstrates that the sinus node transcriptome has a day-night rhythm.

### Functioning circadian clock in the sinus node

**Figure 1:**
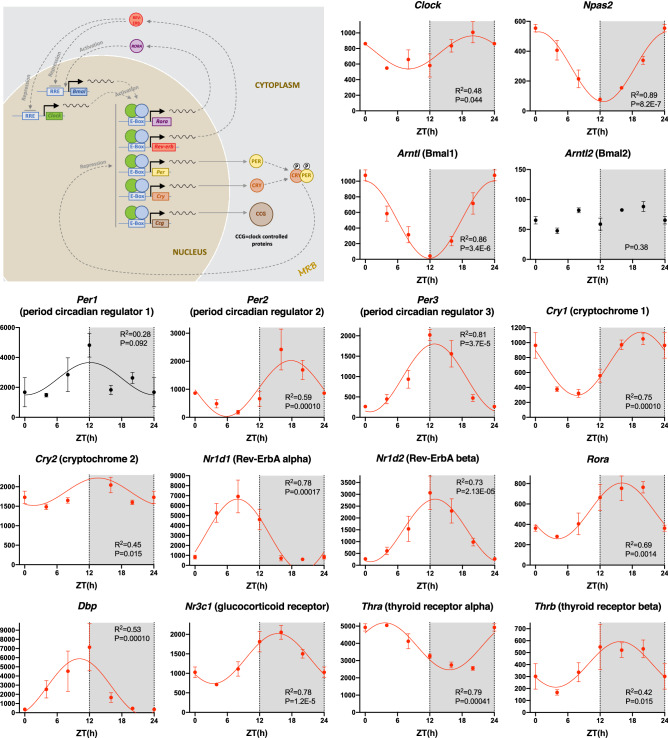
Circadian clock. Abundance (normalised counts) of circadian clock transcripts and some potentially related transcripts is shown over 24 h. The inset shows a schematic diagram of the circadian clock. In this and similar figures: gene name and common name (in parenthesis) given; mean ± SEM transcript abundance shown (n = 3) at six ZT time points over 24 h (24 h data repeat of 0 h data); the permutation-based *P* value (corrected for multiple testing) from JTK Cycle for significance of a day-night rhythm is given; significant transcripts (permutation-based *P* value < 0.05) are shown in red and have been fitted with a sine wave by a least squares fitting method and the R^2^ value given; transcripts showing a trend towards significance (permutation-based *P* value > 0.05 and < 0.1) are shown in black and have again been fitted with a sine wave by a least squares fitting method and the R^2^ value given; non-significant transcripts (permutation-based *P* value > 0.1) are shown in black and have not been fitted with a sine wave.

Figure 1 shows a schematic diagram of the circadian clock. There are two feedback loops. First, CLOCK and BMAL1 proteins dimerise and activate transcription of period (*Per1*, *Per2*, and *Per3*) and cryptochrome (*Cry1* and *Cry2*) genes. PER and CRY proteins then dimerise and repress their own transcription by inhibiting the activity of the CLOCK:BMAL1 dimer. Secondly, CLOCK:BMAL1 dimer activates the transcription of *Nr1d1* (and *Rora*). NR1D1 then represses transcription of *Bmal1* (whilst RORA activates it). All components of the circadian clock are present in the sinus node, including paralogs of *Clock* and *Bmal1* (*Npas2* and *Bmal2*), and all show significant day-night rhythms except *Bmal2* (poorly expressed) and *Per1* (Fig. 1). Table 1 shows that of 19 circadian clock transcripts present, ~ 79% showed a significant day-night rhythm. It is concluded that there is a functioning circadian clock in the sinus node and this could be responsible for the day-night rhythm in other transcripts, selected examples of which are shown below.

**Table 1:** Summary of the percentage of transcripts showing a significant day-night rhythm in different groups and pathways. HGNC, HUGO Gene Nomenclature Committee; MGI, MGI Gene Ontology Browser; RGD, Rat Genome Database.

Group of transcripts	Source of transcripts	Total number of transcripts with > 10 normalised reads	Number showing significant day-night rhythm	Percentage showing significant day-night rhythm
Histone acetyltransferases	HGNC	15	12	80.0%
Circadian clock	Custom selection	19	15	78.9%
Citric acid cycle	RGD	30	20	66.7%
Eukaryotic initiation factors	Custom selection	55	35	63.6%
CaMKII pathway	Kreusser and Backs (2014)	19	12	63.2%
Ca^2+^clock pacemaker mechanism	Custom selection	21	13	61.9%
Glycolysis pathway	RGD	54	32	59.3%
Autonomic receptors and their pathways	Custom selection	31	18	58.1%
Mitochondrial transporters (Slc25 transcripts)	–	40	23	57.5%
Histone deacetylases	Wikipedia	17	9	52.9%
RNA degradation pathway	Houseley and Tollervey^79^	38	20	52.6
Transcription factors	Custom selection	703	347	49.4%
Fatty acid β-oxidation	MGI	61	30	49.2%
Ubiquitin and proteasome	Custom selection	46	22	47.8%
G-protein α β and γ subunits	HGNC	25	12	48.0%
All transcripts	–	16,387	7134	43.5%
Electron transport chain	MGI	74	32	43.2%
RNA polymerases	Custom selection	26	11	42.3%
Solute carriers (Slc transcripts)	–	278	114	41.0%
Ca^2+^ ion transport	MGI	348	128	36.8%
G-protein coupled receptors (Gpr genes)	–	52	17	32.7%
Extracellular matrix	MGI	378	122	32.3%
Ion channel subunits	HGNC	199	64	32.2%
Gap junction subunits	Custom selection	13	2	15.4%
microRNAs	–	–	67	–

Circadian clocks in peripheral tissues are known to be entrained to the master circadian clock in the suprachiasmatic nucleus via neurohumoral regulation. Potential entrainment signals have been suggested to be plasma glucocorticoids^25^ and thyroid hormone^26^ as well as the autonomic nervous system^25^. It is therefore interesting that transcripts for glucocorticoid and thyroid hormone receptors (Fig. 1) as well as receptors for autonomic transmitters (see below) all showed significant day-night rhythms.

### Day-night rhythm in pacemaker genes

**Figure 2:**
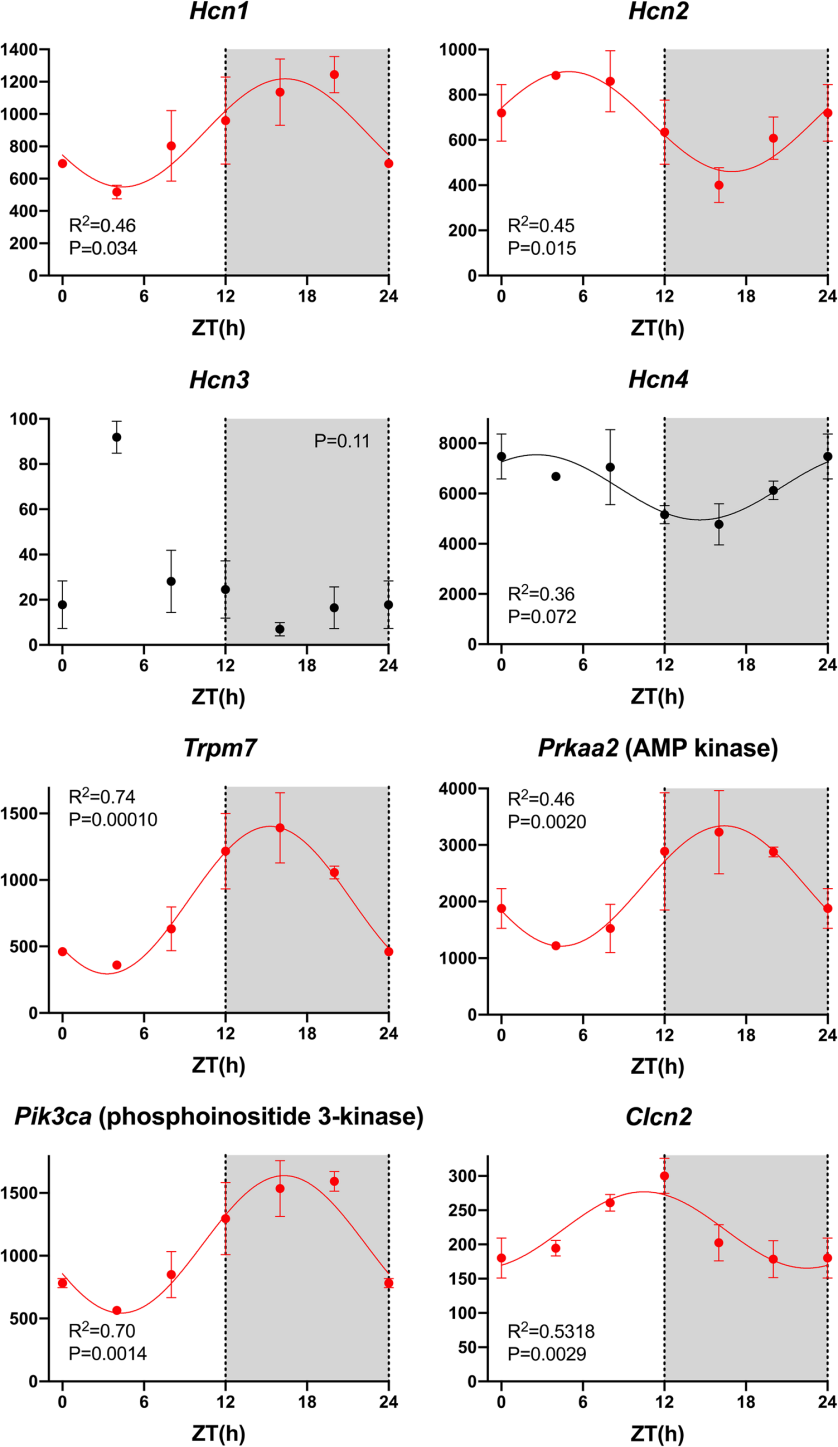
Membrane clock pacemaker mechanism. Abundance (normalised counts) of pacemaker ion channel transcripts and some potentially related transcripts is shown over 24 h. See Fig. 1 legend for further details.

**Figure 3:**
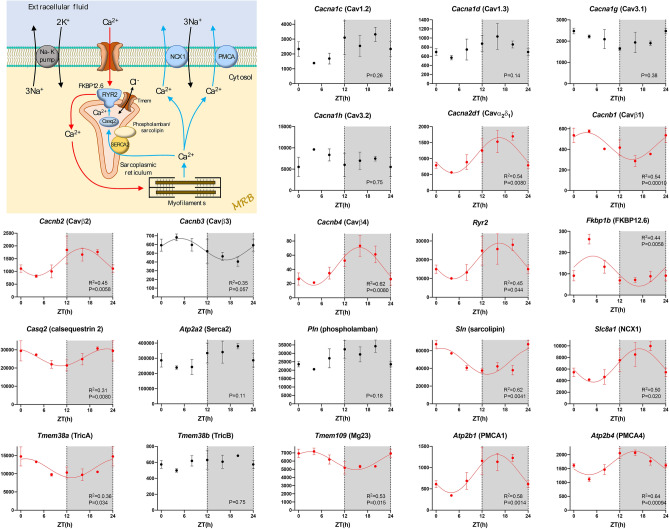
Ca^2+^ clock pacemaker mechanism. Abundance (normalised counts) of Ca^2+^ clock transcripts is shown over 24 h. The inset shows a schematic diagram of the Ca^2+^ clock. See Fig. 1 legend for further details.

Pacemaking in the sinus node is widely regarded as the result of two mechanisms, the membrane and Ca^2+^ clocks^27,28^. The main contributor to the membrane clock are the HCN (funny) channels. All four *Hcn* isoform transcripts were present and *Hcn4* >  > *Hcn1*≈*Hcn2* >  > *Hcn3* as expected (Fig. 2). *Hcn1* and *Hcn2* showed significant day-night rhythms, whereas *Hcn3* did not and *Hcn4* showed a trend towards a rhythm (P = 0.072) (Fig. 2). Using quantitative PCR, we have previously shown a qualitatively similar, but significant, day-night rhythm in *Hcn4* in the mouse sinus node^13^. TRPM7 (a divalent-permeant channel-kinase)^29^, AMP kinase^30^ and phosphoinositide 3-kinase^31^ have been shown to regulate funny channels and/or current, and their transcripts all showed significant day-night rhythms (Fig. 2). CLCN2 is an ion channel responsible for a Cl^-^ current that functions in a similar way to the funny current and has been shown to contribute to pacemaking^32^, and *Clcn2* too showed a significant rhythm (Fig. 2). Figure 3 shows a schematic diagram of intracellular Ca^2+^-handling and, therefore, the main components of the Ca^2+^ clock: surface membrane Ca^2+^ channels and their accessory subunits, the sarcoplasmic reticulum (SR) Ca^2+^ release channel (RYR2), FKBP12.6, the SR Ca^2+^ pump (SERCA2), phospholamban/sarcolipin, calsequestrin 2, and the Na^+^-Ca^2+^ exchanger (NCX1). For completeness, the diagram also shows SR Cl^-^ channels (which balance charge associated with Ca^2+^ movements in and out of the SR)^33^ and surface membrane Ca^2+^ pumps (PMCAs) (Fig. 3). Not all, but many, of the corresponding transcripts showed a significant day-night rhythm (Fig. 3). Two of the most important players in the Ca^2+^ clock are RYR2 and NCX1 and transcripts for both showed prominent and significant day-night rhythms (Fig. 3). Table 1 shows that of 21 transcripts involved in the Ca^2+^ clock, ~ 62% showed a significant day-night rhythm. It is concluded that there is a day-night rhythm in transcript expression for many of the key players involved in pacemaker activity in the sinus node.

### Other ion channels, gap junction channels and the Na^+^-K^+^ pump

The HGNC website provides a list of 328 ion channel subunits, of which 199 were present in the RNAseq dataset. Of these ~ 32% showed a significant day-night rhythm—listed in Table S1. Examples are shown in Fig. S2. One example, the TASK-1 channel, is highly abundant and its transcript showed a significant day-night rhythm and yet the role of this channel in the sinus node is not known. In the case of surface membrane ion channels, the day-night rhythm could potentially impact pacemaking. Transcripts for twelve gap junction subunits were identified; *Gja5* (Cx40) showed a significant day-night rhythm and *Gja1* (Cx43; *P* = 0.092) and Cx45 (*Gjc1*; *P* = 0.072) showed a trend towards one (Fig. S3). In addition, TMEM65, which interacts with and functionally regulates Cx43^34^, showed a significant day-night rhythm (Fig. S3). Gap junctions, by controlling the interaction of the pacemaking sinus node with the hyperpolarized non-pacemaking neighbouring atrial muscle, affect pacemaking^35^ and, therefore, a day-night rhythm in connexin protein could potentially impact pacemaking. Ionic currents are dependent on ionic gradients across the cell membrane set up by the Na^+^-K^+^ pump. There are three Na^+^-K^+^ pump α-subunits and transcripts for all three tended to or showed a significant day-night rhythm (Fig. S4). The Na^+^-K^+^ pump is regulated by phospholemman^36^ and its transcript too showed a significant day-night rhythm (Fig. S4). Is Na^+^-K^+^ pump expression (and therefore activity) greater during the awake period when the heart rate is higher in order to counter the greater movement of ions through ion channels across the cell membrane?

### Autonomic receptors and downstream pathways

**Figure 4:**
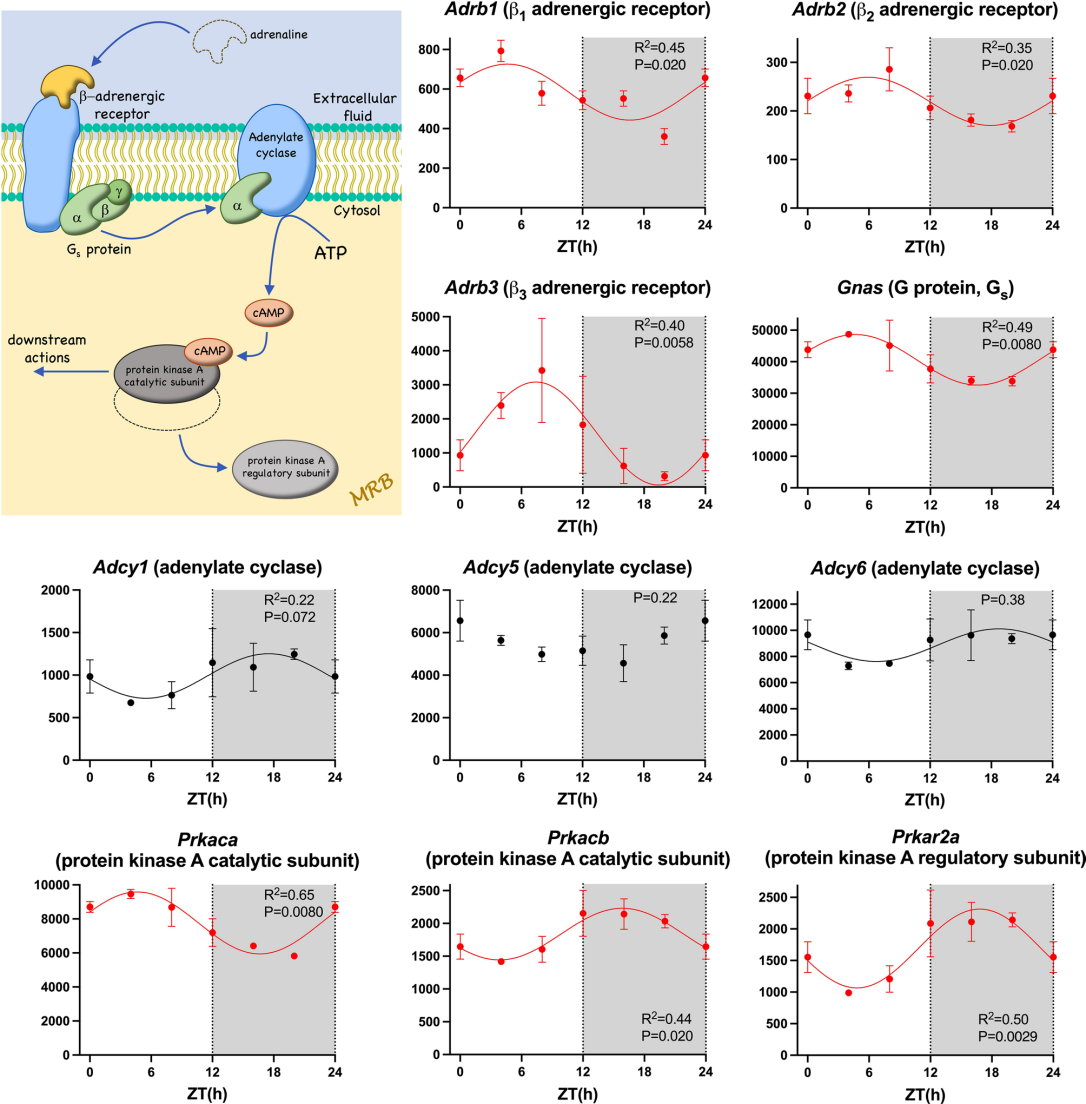
β-adrenergic receptor pathway. Abundance (normalised counts) of β-adrenergic receptor pathway transcripts is shown over 24 h. The inset shows a schematic diagram of the β-adrenergic receptor pathway. Transcripts for two other protein kinase A subunits, *Prkar1b* and *Prkar2b*, also showed a significant circadian rhythm (data not shown). See Fig. 1 legend for further details.

Heart rate is regulated by the autonomic nervous system. The day-night rhythm in heart rate is widely attributed to a day-night rhythm in the autonomic innervation of the heart: high vagal tone during sleep resulting in rapid changes in ionic conductances in the sinus node and thus heart rate. However, curiously, transcripts for some of the autonomic receptors and their immediate downstream mediators showed a day-night rhythm. Binding of catecholamine to the α_1_ adrenergic receptor activates phospholipase C via a G protein (G_q_) and this ultimately results in the activation of the IP_3_ receptor and protein kinase C (Fig. S5). Of the three α_1_ adrenergic receptor transcripts expressed, the α_1B_ adrenergic receptor transcript was the most abundant and this showed a significant day-night rhythm (Fig. S5). Many of the downstream transcripts including transcripts for the α subunit of G_q_, phospholipase C, two IP_3_ receptor isoforms and protein kinase C, also showed a significant day-night rhythm (Fig. S5). Binding of catecholamine to the β adrenergic receptor activates protein kinase A via a G protein (G_s_) and adenylate cyclase. β_1_, β_2_ and β_3_ adrenergic receptor transcripts were present; surprisingly, whereas the β_1_ adrenergic receptor is considered the most important in the heart, the β_3_ adrenergic receptor transcript was the most abundant. Transcripts for β_1_, β_2_ and β_3_ adrenergic receptors, the α subunit of G_s_, and the catalytic subunit of protein kinase A showed a significant day-night rhythm (Fig. 4). Binding of ACh to the M2 receptor activates the ACh-activated K^+^ channel via a G protein (G_i_), and transcripts for the receptor, the α subunit of G_i_, and one of the two subunits making up the channel (Kir3.1) showed a significant day-night rhythm (Fig. S6). The same pathway is activated by the adenosine A1 receptor^37^ and this too showed a significant day-night rhythm (Fig. S6).

### Signalling pathways

There are many signalling pathways in the heart and just a few examples were investigated. Ca^2+^/calmodulin-dependent protein kinase II (CaMKII) plays an important role in the heart and the sinus node. Whereas acute activation of CaMKII results in phosphorylation of downstream targets by the kinase^38^, chronic activation results in transcriptional remodelling^39^. For example, constitutive activation of CaMKII (by oxidation) in heart failure is reported to be responsible for sinus node disease common in heart failure^40^. Figure S7 shows a schematic diagram of the pathway (based on Kreusser and Backs^39^) by which CaMKII regulates gene transcription. Transcripts for CaMKII (*Camk2d*) and many of its downstream mediators showed a significant rhythm (Fig. S7). Mitogen-activated protein kinases (MAP kinases) are involved in cardiac development, physiological adaptation and pathological manifestation^41^. They act on ion channels^42^. They form a three-tiered kinase cascade in which a MAP kinase kinase kinase activates a MAP kinase kinase, which in turn activates a MAP kinase^41^. Transcripts for some kinases in all three tiers of the cascade showed significant rhythms (Figs. S8-S10). Nitric oxide (NO) is an important regulator of the cardiovascular system and in part NO is derived from NO synthases (NOSs)^43^. Of the three NOS transcripts present, *Nos3* (inducible NOS, iNOS) showed a significant day-night rhythm (data not shown). Any signalling pathway impacting surface membrane ion channels or intracellular Ca^2+^ handling has the potential to affect pacemaking.

### Myofilaments

Some of the transcripts for the contractile apparatus of the sinus node myocyte showed a significant day-night rhythm, including transcripts for myosin light chain 4 and titin, which are linked to sick sinus syndrome^44,45^. In addition, transcripts for actin, tropomyosin 1 and troponin I showed significant day-night rhythms (Fig. S11).

### Metabolism

**Figure 5:**
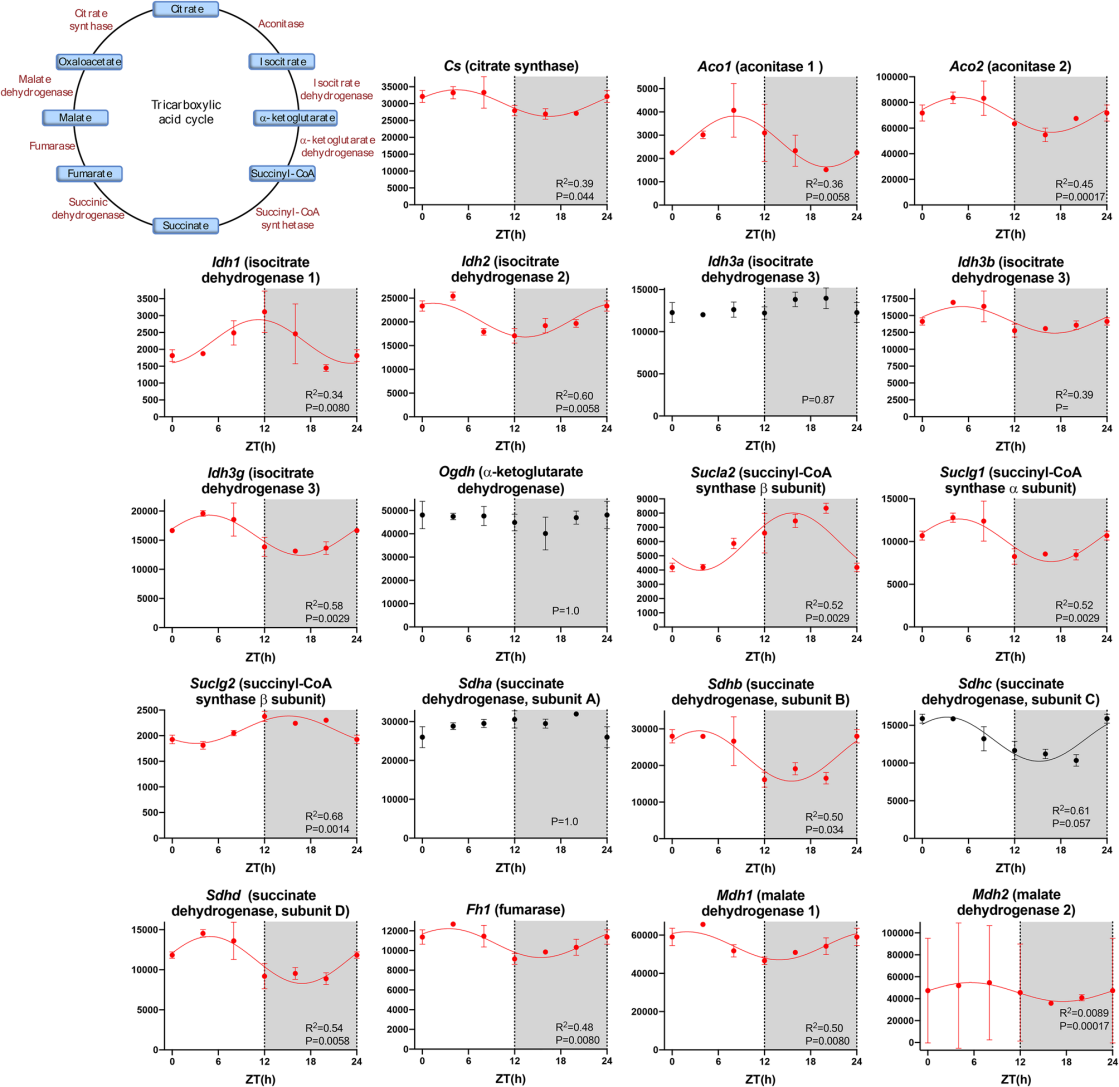
Citric acid cycle. Abundance (normalised counts) of citric acid cycle transcripts is shown over 24 h. The inset shows a schematic diagram of the citric acid cycle. See Fig. 1 legend for further details.

It is well known that metabolism shows a day-night rhythm, including in the heart e.g.^23^. The RNAseq data suggests that this is also true of the sinus node. ~ 70–90% of cardiac ATP is produced by the oxidation of fatty acids, which are transported into the mitochondria as acyl-CoA^46^. Carnitine palmitoyltransferases I and II and translocase are involved in the transport and transcripts for carnitine palmitoyltransferase II and translocase showed a significant day-night rhythm (Fig. S12). The long-chain acyl-CoA enters the fatty acid β-oxidation pathway. ~ 49% of the transcripts for the fatty acid β-oxidation pathway showed a significant day-night rhythm (Table 1)—examples are shown in Fig. S13. The acetyl-CoA then enters the citric acid cycle to generate ATP. Glycolysis is the metabolic pathway that converts glucose into pyruvate; the pyruvate is then converted to acetyl-CoA, which again then enters the citric acid cycle to generate ATP. ~ 59% of the transcripts for the glycolysis pathway showed a significant day-night rhythm (Table 1)—examples are shown in Fig. S14. ~ 67% of the transcripts for the citric acid cycle itself showed a significant day-night rhythm (Table 1)—examples are shown in Fig. 5. The NADH generated by the citric acid cycle is fed into the oxidative phosphorylation pathway—involving the electron transport chain—to form ATP. ~ 43% of the electron transport chain transcripts showed a significant day-night rhythm (Table 1)—examples are shown in Fig. S15. The day-night rhythm in metabolism has the potential to impact pacemaking, because AMP kinase (Fig. 2) both conserves cellular energy homeostasis and also affects the heart rate via the pacemaker funny current carried by HCN channels^30^. For example, ATPIF1 is an inhibitor of mitochondrial ATPase, the engine of oxidative phosphorylation, and consequently a regulator of energy metabolism^47^. ATPIF1 has a link to AMP kinase^48^ and *Atpif1* showed a trend of a day-night rhythm (*P* = 0.072; Fig. S15).

### Extracellular matrix

**Figure 6:**
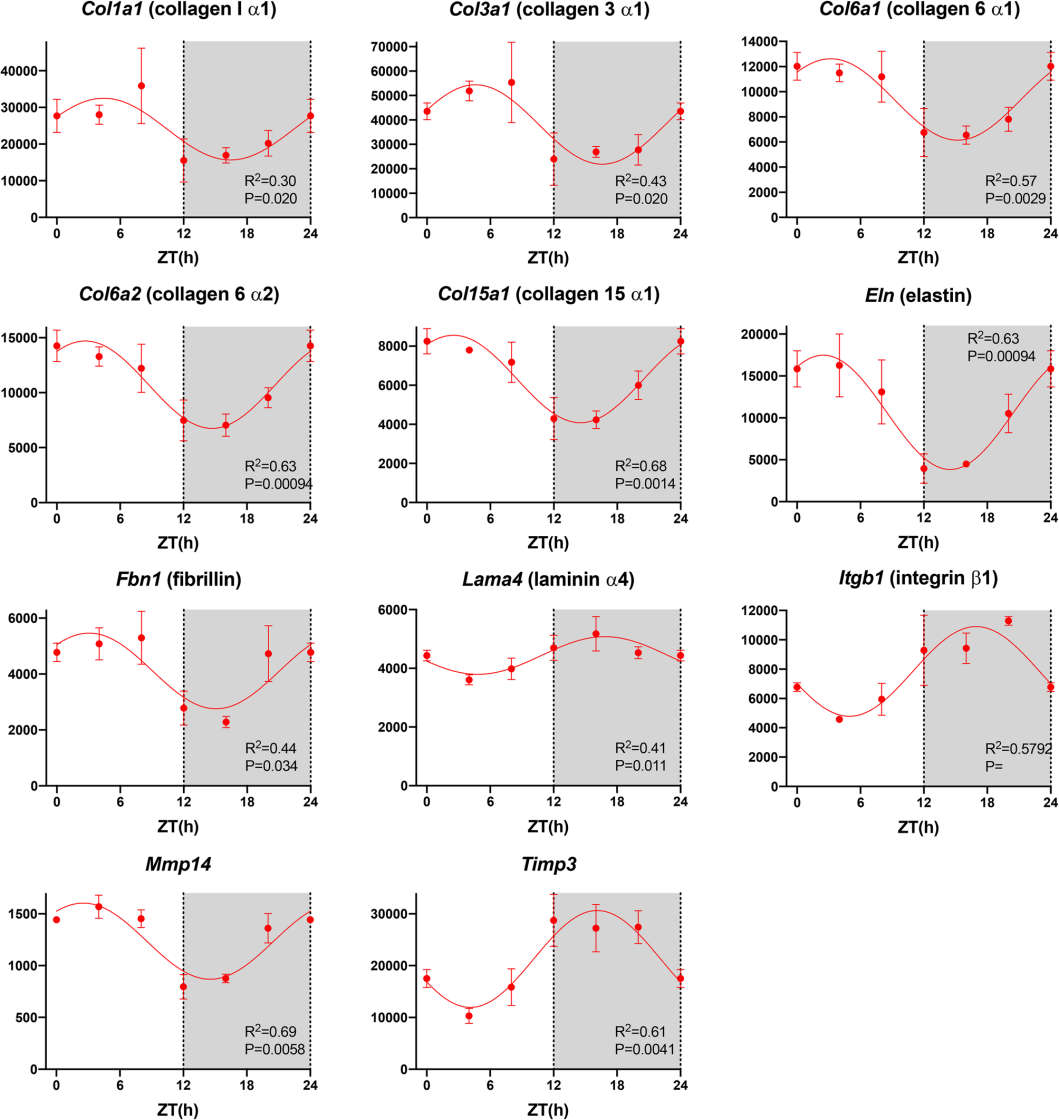
Extracellular matrix. Abundance (normalised counts) of example extracellular matrix transcripts is shown over 24 h. See Fig. 1 legend for further details.

Of 378 transcripts involved in the extracellular matrix, ~ 32% showed a significant day-night rhythm (Table 1)—examples are shown in Fig. 6. The extracellular matrix, as well as being essential for the structural integrity of the heart, is a load that the contractile apparatus must deform in the cardiac cycle—it is therefore an energetic cost. Could the extracellular matrix change from day to night as the heart rate and cardiac output change to obtain the necessary structural integrity at the least energetic cost? Proliferation of the extracellular matrix has long been linked to sinus node dysfunction.

### Immune system

The immune system is known to show a circadian rhythm^49^. The class I major histocompatibility complex (HMC) of a tissue presents self-antigens to cytotoxic T-cells of the immune system and ultimately prevents the animal’s immune system targeting its own cells, whereas the class II MHC presents pathogen-derived proteins ultimately resulting in the elimination of infected cells by the immune system. The class II MHC is linked to heart failure^50,51^. In the sinus node, two transcripts involved with the class I MHC (*H2-k1* and *H2-q10*^52^), a transcript for a class I MHC-like molecule (*Cd1d1*^53^), two transcripts potentially involved with the class I MHC (*Rpp21* and *Trim39*) and one transcript (*H2-dmb2*^52^) involved with the class II MHC showed a significant day-night rhythm (Fig. S16). Respiratory (or oxidative) burst is the rapid release of reactive oxygen species (ROS), superoxide anion, and hydrogen peroxide. Macrophages and neutrophils are especially implicated in the respiratory burst. They are phagocytic, and the respiratory burst is vital for the subsequent degradation of internalised bacteria or other pathogens. This is an important aspect of immunological defence. There was a significant day-night rhythm in *Cybb* (NOX2) encoding the major component of NADPH oxidase, which plays a key role in the respiratory burst (Fig. S16). Interleukin 33 is an alarmin (type of cytokine) and is released under conditions of stress to induce protective measures^54^. For example, it has been shown to antagonise cardiac hypertrophy and remodelling in mice subject to transverse aortic constriction^54^. The transcript for interleukin 33 (*Il33*) showed a significant day-night rhythm (Fig. S16) raising the possibility that the heart’s resistance to stress may also show a day-night rhythm. Heart disease is frequently associated with sinus node dysfunction^55^ and the immune system has been linked to the adverse remodelling of the diseased heart^50^. Other transcripts linked to the immune system also showed a significant day-night rhythm and are illustrated in Fig. S17 or listed in Table S2.

### GWAS-identified genes associated with resting heart rate

Various genome-wide association studies (GWAS) have been carried out to identify genetic variants (and therefore genes) affecting the resting heart rate. The majority of these genes have not previously been identified as involved in pacemaking. Of the genes identified by these GWAS studies, transcripts for 46 showed a significant day-night rhythm (P < 0.05) and, therefore, could potentially contribute to a day-night rhythm in pacemaking—the transcripts are listed in Table S2 together with transcripts which showed a trend of a day-night rhythm (0.1 > *P* > 0.05). In most cases, the nature of the relationship between the gene and pacemaking is unknown. In a few cases, there is a plausible link to pacemaking (cAMP-dependent protein kinase type II-alpha regulatory subunit, *Prkar2a*—Fig. 4; muscarinic M2 receptor, *Chrm2*—Fig. S6; acetylcholine esterase, *Ache*—Fig. S6; titin, *Ttn*—Fig. S11^44^; *Hcn4*—Fig. 2; Cx43, *Gja1*—Fig. S3; desmoplakin, *Dsp*—Fig. S3^56^). Some genes are related to ion channels, ion transport, receptors or a signalling pathway (*Alg10*, *Slc12a9*, *Calcrl*, *Gng11*, *Map3k10*) and, therefore, a relationship with pacemaking is not implausible. Some genes are involved in transcription, translation or degradation of mRNA/protein and could potentially be involved with pacemaker genes (*Mkln1*, *Klhl42*, *Canx*, *Ppargc1a*, *Tbx20*, *Rnf220*, *Ppil1*, *Ddx17*, *Srrt*, *Srebf1*, *Ufsp1*, *Cby1*, *Cdc23*). Intriguingly, one of the genes, *Gtpbp1*, promotes degradation of target mRNA species and plays a role in the regulation of circadian mRNA stability^57^. In some cases, a link with pacemaking can be speculated on: *Met* is a transcript for a receptor tyrosine kinase, which is known to target phosphoinositide 3-kinase^58^, which in turn is known to target HCN4^31^. *Ephb4* is a transcript for another receptor tyrosine kinase, and could it act in a similar way? Inositol hexakisphosphate kinase 1 (*Ip6k1*) has a link to AMP kinase^59^, which is known to regulate HCN4^30^.

### Transcription, translation, and mRNA and protein degradation

**Figure 7:**
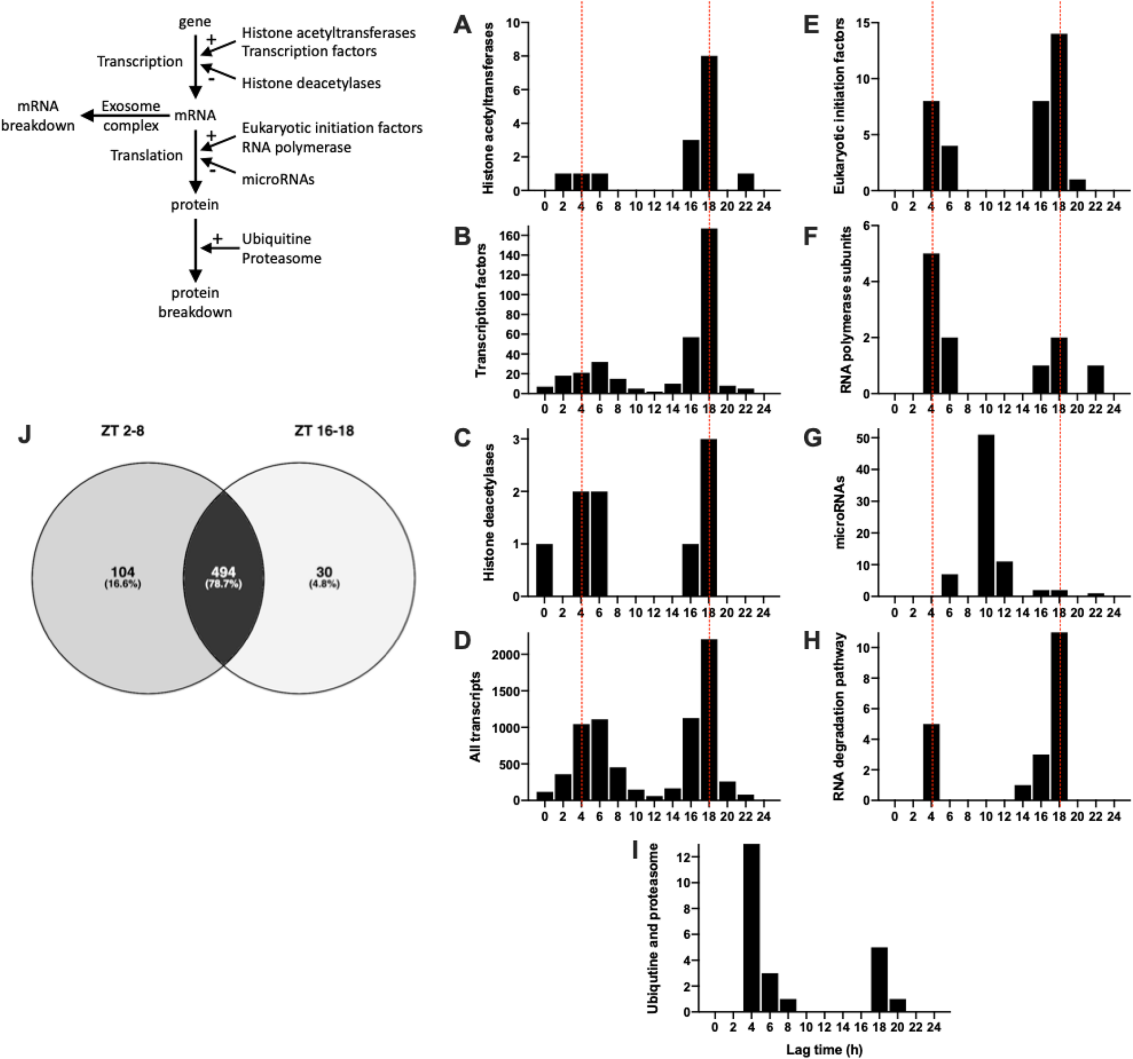
Transcription, translation, and mRNA transcript and protein breakdown. (**A**–**I**), histogram of lag times (times of peak transcript abundance) of different groups of transcripts involved in transcription, translation, and mRNA transcript and protein breakdown. (**J)**, Venn diagram of pathways involving the transcripts peaking during the day and night; data were analysed through the use of IPA (QIAGEN Inc., https://www.qiagenbioinformatics.com/products/ingenuitypathway-analysis)^103^. Inset, schematic diagram of the cycle of transcription, translation, and RNA and protein degradation.

**Figure 8:**
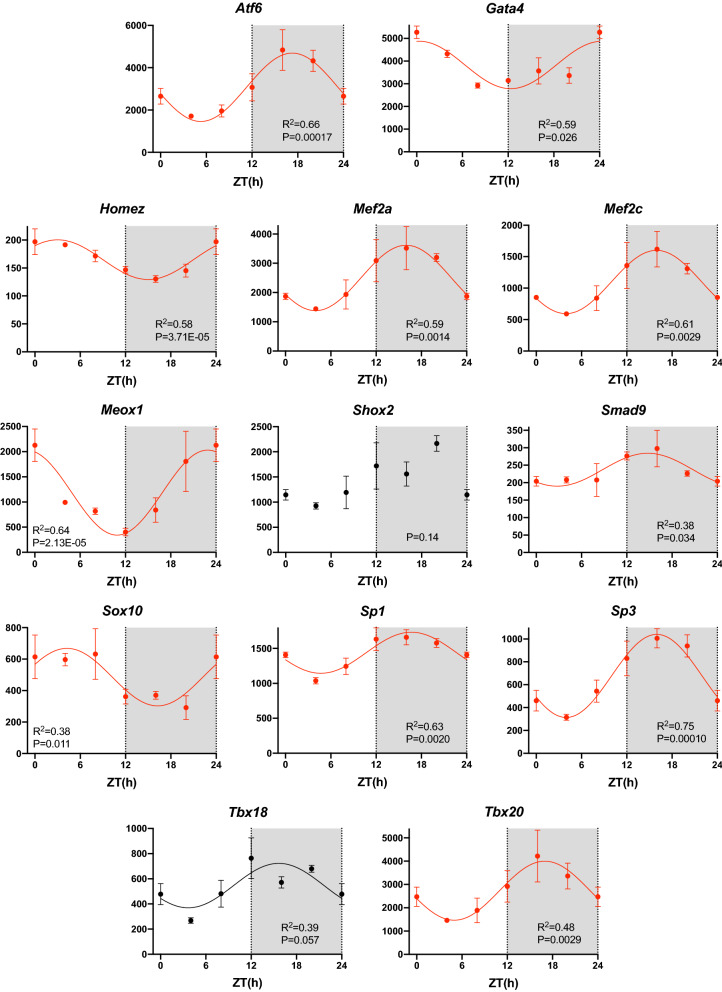
Transcription Factors. Abundance (normalised counts) of example transcription factor transcripts is shown over 24 h. See Fig. 1 legend for further details.

Figure 7D shows the lag time of the 7134 transcripts showing a significant day-night rhythm. The lag time corresponds to the maximum expression during the 24 h period. There are two lag time peaks, one in the day (~ ZT 4–6) and one at night (~ ZT 18), the most prominent being the one at night (Fig. 7D). This suggests that there are two peaks in the process of transcription. Transcription is controlled by histone acetyltransferases (HATs), which make chromatin accessible for transcription, and transcription factors, which drive transcription. Fifteen HAT transcripts were identified and 80% showed a significant day-night rhythm (Table 1). Recently, Zhou et al*.*^60^ have published an atlas of 941 mouse transcription factors. Of these, transcripts for 703 were identified in the sinus node and ~ 49% showed a significant day-night rhythm (Table 1)—examples are shown in Fig. 8. The chosen examples include transcripts for transcription factors known to be involved in determining the sinus node pacemaking phenotype (*Tbx18*^61^), sinus node development (*Mef2c*^62^), cardiac development (*Homez*, *Sp3*, *Gata1*^63–65^), cardiac development and adult function (*Tbx20*^66^), cardiac development and possibly the circadian clock (*Nrd1d2*^67,68^) and cardiac disease (*Atf6*, *Meox1*^69,70^). Sp1 is a regulator of many cardiac genes including Serca2^71^ and genes involved in metabolism^72^. Smad signalling has been linked to cardiac remodelling following myocardial infarction^73^. Sox10-positive cardiomyocytes of neural crest origin contribute to myocardial regeneration in the Zebrafish^74^. Mef2 directly targets HCN4^75^. Shox2 is essential for the differentiation of cardiac pacemaker cells^76^. The oscillating HAT and transcription factor transcripts peaked at approximately the same time during the day and night (~ ZT 4 and 18; Fig. 7A,B) as all transcripts (Fig. 7D). Could these be responsible for the two peaks in transcription (Fig. 7D)? Histone deacetylases (HDACs) close chromatin to transcription—of 17 HDAC transcripts identified, ~ 53% showed a significant day-night rhythm (Table 1). Again HDAC transcripts showed the same two peaks (Fig. 7C). This is not unexpected—if transcription occurs primarily in two relatively brief spurts, the HATs and HDACs are expected to be present at roughly the same times. Intuitively, HDACs are expected to peak later than HATS to provide a time window when chromatin is accessible for transcription, but perhaps such a time window will only be evident with more frequent sampling than once every 4 h. Translation is governed by eukaryotic initiation factors (which guide transcripts to the ribosomes) and RNA polymerases. Of 55 transcripts for eukaryotic initiation factors identified, ~ 66% showed a significant day-night rhythm (Table 1). Of 26 RNA polymerase transcripts identified, ~ 42% showed a significant day-night rhythm (Table 1). It was only transcripts for RNA polymerase 2 (responsible for transcribing precursors of mRNA and most snRNA and microRNA) and 3 (responsible for transcribing housekeeping genes: 5S ribosomal RNA, tRNA and other small RNAs) which showed day-night rhythms—RNA polymerase 1 (responsible for transcribing ribosomal RNA) did not. Once again, transcripts for eukaryotic initiation factors and RNA polymerases showed the same two peaks (Fig. 7E,F). microRNAs are short non-coding RNAs—they downregulate the expression level of transcripts (and thereby downregulate translation) or they directly downregulate translation of transcripts. In a separate study, we measured the expression of microRNAs at four time points at 6 h intervals over 24 h using qPCR^77^. 74 microRNAs showed a significant day-night rhythm and unlike transcription factors and all transcripts showed a single peak at ~ ZT 10 (Fig. 7G). microRNAs are, therefore, in antiphase to transcription factors etc. This is not unexpected, because whereas transcription factors upregulate transcription/translation, microRNAs downregulate them.

Two peaks in transcripts during the day and at night have been seen before in mouse heart (presumably dominated by ventricular muscle) by Zhang et al.^20^ However, there are important differences between the two studies: in ventricle, 1335 oscillating transcripts were identified, which Zhang et al.^20^ reports is consistent with the 3–10% of the transcriptome oscillating in different tissues. In contrast, in the present study of the sinus node 7134 oscillating transcripts (~ 44% of the transcriptome) were identified. This is a much higher percentage than in ventricle and other tissues and reasons for this are speculated on in the Discussion. In addition, the segregation into two peaks is much more marked in sinus node (Fig. 7D) than in ventricle^20^. In ventricle, Zhang et al.^20^ argued that the transcripts in the two peaks were different in nature and the biphasic distribution of transcripts was under the control of the transcription factor, Klf15. However, no evidence of this was found in the sinus node: the types of transcripts said to be restricted to one peak in ventricle were not restricted to the same peak in the sinus node, and analysis of all significantly rhythmic transcripts in the two peaks in the sinus node by Ingenuity Pathway Analysis (IPA) software revealed no pattern and the enriched pathways in the two peaks were mostly similar (494 of 628 pathways enriched in the peaks were common to both; Fig. 7J). Furthermore, *Klf15*, did not show a significant day-night rhythm in the sinus node (P = 0.22; data not shown). There is further discussion about the phasing of transcripts in the Supplementary Information.

The potential targets of the 74 oscillating microRNAs were investigated using IPA, which utilises TargetScan predictions, experimentally validated TarBase and miRecords targets and manual curations from the literature. Experimentally observed targets as well as high and moderate confidence predictions were considered, although it is acknowledged that there is some likelihood of false positive predictions resulting from these analyses. Oscillating microRNAs were screened against the rhythmic transcripts using the microRNA target filter function in IPA, with a mouse species filter applied. Of the 7134 oscillating transcripts, 4982 (70%) were predicted to be targeted by 57 of the oscillating microRNAs.

It is assumed that the day-night rhythms in transcripts are the result of the oscillating transcription factors and microRNAs. In total, 16,387 transcripts were detected and 703 transcription factors were detected. In a previous study we have detected 715 microRNAs in the mouse sinus node^78^. Therefore, the ratio of transcription factors:transcripts is ~ 1:23 and the ratio of microRNAs:transcripts is 1:23. There are 7134 oscillating transcripts, 347 oscillating transcription factors and a minimum of 74 oscillating microRNAs—therefore the ratio of oscillating transcription factors:oscillating transcripts is ~ 1:21 and the ratio of oscillating microRNAs:oscillating transcripts is ~ 1:96.

At steady-state, RNA and protein degradation has to match transcription and translation. The many pathways of RNA degradation were reviewed by Houseley and Tollervey^79^; 38 components of these pathways were identified and ~ 53% showed a significant day-night rhythm (Table 1). Proteins are tagged for degradation by ubiquitination catalysed by ubiquitin ligases. Once ubiquinated, the protein is degraded by the proteasome. 47 transcripts for this pathway were identified and ~ 49% showed a significant day-night rhythm (Table 1). Once again these showed the same two peaks (Fig. 7H,I).

## Discussion

The sinus node has two clocks, the membrane and Ca^2^^+^ clocks, operating on a time scale of seconds, and which are responsible for the rhythmic beating of the heart. For the first time, this study has shown that the sinus node has another clock, the circadian clock, operating on a time scale of days and which could be responsible for, or at least involved in, a rhythmic change in the transcriptome of the sinus node. ~ 44% of the transcriptome of the sinus node is changing in a day-night manner and the day-night rhythm is all-pervasive affecting all systems looked at including the membrane and Ca^2+^ clocks, neurohumoral receptors, important signalling pathways, metabolism and extracellular matrix. The interested reader is likely to find other systems affected—a list of all transcripts together with the permutation-based *P* value from JTK Cycle for a day-night rhythm is available as part of the Supplementary Data (AllTranscripts.xlsx).

### Day-night rhythm in heart rate

Based on heart rate variability and autonomic blockade, the day-night rhythm in heart rate is currently attributed to changes in the autonomic innervation of the heart and in particular to high vagal tone at night in the case of the human^13^. According to this hypothesis, ACh released from vagal nerve endings binds to muscarinic M2 receptors and activates the ACh-activated K^+^ channel, and this causes the slowing of heart rate at night. However, we have argued that heart rate variability cannot be used to measure autonomic innervation of the heart^80^, and data from autonomic blockade has been reported to both block and have no discernible effect on the day-night rhythm in heart rate^13^. However, although there is doubt concerning the evidence for a day-night rhythm in autonomic innervation of the heart, it is clear that there is a day-night rhythm in the plasma level of catecholamine (presumably coming from the adrenal medulla under the action of the sympathetic nervous system), which is higher during the day in the human^81^. Therefore, it is possible that a day-night rhythm in the autonomic nervous system is responsible for the day-night rhythm in heart rate via rapid regulation of ionic conductances. This study does not resolve this controversy, but it does show that at the transcript level the two major pacemaking mechanisms of the sinus node, the membrane and Ca^2+^ clocks, show a profound day-night rhythm (Figs. 2,3). Therefore, it is possible that there is a day-night rhythm in pacemaking as a result of changes in gene transcription in the sinus node. In another study, we have shown that there is indeed an intrinsic day-night rhythm in both funny current density and pacemaking^82^. However, although an intrinsic day-night rhythm in pacemaking could be responsible for the day-night rhythm in heart rate, it may only be responsible for a day-night rhythm in ‘pacemaker reserve’ so that the sinus node is prepared to deliver higher heart rates during the awake period when called upon to do so by the autonomic nervous system. The final answer to the controversy of whether the day-night rhythm in heart rate is the result of the post-translational regulation of ion channels by the autonomic nervous system or transcriptional changes is unlikely to be simple. This study has shown that there is a day-night rhythm in expression of autonomic receptors (adrenergic and muscarinic receptors; Figs. 4, S5 and S6) and, therefore, there may be a day-night rhythm in the responsiveness to the autonomic receptor stimulation. Another possibility is that the autonomic nervous system is involved, but in a different way to that originally conceived: Tong et al.^83,84^ have shown that autonomic blockade abolishes the day-night rhythm in the expression of various K^+^ channels and connexin subunits in the ventricles (see below for further comment).

### Day-night rhythm in the transcriptome

Based on the use of Affymetrix GeneChip oligonucleotide arrays, Storch et al.^21^ estimated that about 10% of the mouse liver transcriptome shows a significant day-night rhythm; they detected 4,805 transcripts in the liver of which 575 transcripts oscillated with a day-night rhythm, but they attributed 16% of these to noise rather than a genuine day-night rhythm. Using Affymetrix GeneChip oligonucleotide arrays, Martino et al.^18^ detected 12,488 transcripts in mouse heart (likely to be exclusively or mainly the ventricles based on tissue mass), of which 1,634 (~ 13%) showed a significant day-night rhythm (based on use of COSOPT) during a normal 12 h light:12 h dark lighting regime. In a more recent study, using RNAseq, Zhang et al.^20^ identified 1,335 transcripts (based on use of JTK Cycle) showing a significant day-night rhythm in mouse heart (presumably ventricle) during a normal 12 h light:12 h dark lighting regime. In a variety of studies on mouse heart (presumably ventricle), 6–13% of the transcriptome has been reported to vary in a day-night manner^18–21^. Using both RNAseq and Affymetric MoGene oligonucleotide arrays, Zhang et al.^19^ looked at the day-night rhythm in the transcriptome in 12 mouse organs; they reported that the transcripts oscillating varied from 3% in the hypothalamus, 6% in the heart and 16% in the liver. In the present study, 16,387 transcripts were selected, of which 7134 (~ 44%) showed a significant day-night rhythm and the fraction of oscillating genes is clearly much higher than in other studies. The data from the present study are robust and it is concluded that the difference is a tissue difference. The reason why the fraction of transcripts under day-night control is large in the sinus node can only be speculated on. One possibility is the importance of heart rate for an organ that is continuously beating every ~ 1 s throughout life; cardiac output is primarily determined by variation in heart rate rather than by variation in stroke volume. The pacemaker activity of the sinus node has to be tuned for higher heart rates during the day in the human and this perhaps not only involves a day-night rhythm in the membrane and Ca^2+^ clock pacemaker mechanisms, but also in closely associated systems involving receptors and signalling for example. Presumably because the heart is continuously active, O_2_ utilisation per 100 g of tissue is highest for the heart. Because work carried out by the heart is primarily determined by the heart rate, O_2_ utilisation will be primarily determined by the heart rate. Perhaps for this reason, pacemaking and metabolism have to be controlled together including in a day-night manner and there have to be links between the two; AMP kinase could be one of these links^30,85^. For a similar reason, perhaps pacemaking and the extracellular matrix have to be controlled together to tune the extracellular matrix for the higher heart rate and pressures during the day in the human.

### Day-night rhythm in transcription, translation, and mRNA and protein degradation

Transcripts changing in a day-night manner peaked either during the day (~ ZT 4–6) or during the night (ZT 18) (Fig. 7D). This pattern has been seen before: in mouse liver and heart (presumably ventricle) there were peaks at ZT 6–14 and ZT 20^21^, and in other studies of mouse heart (presumably ventricle) there were peaks at ZT 1 and ZT 19^18^ or ~ ZT 1 and ZT 17^20^. However, in all of these cases the day-time peak was larger than the night-time peak, whereas the opposite was true in the case of the sinus node (Fig. 7D). The present study has shown the likely immediate cause of this pattern. Transcripts for the transcription apparatus (HATs, transcription factors, HDACs) peaked at ~ ZT 4–6 and then at ZT 18 and this ultimately was likely to be responsible for the peak in transcripts at these two time points (Fig. 7). Transcripts for the translation apparatus (eukaryotic initiation factors, RNA polymerases) also peaked at the same time points and therefore generation of protein is expected to peak at the same time points (Fig. 7). Finally, transcripts for the apparatus for the breakdown of both transcripts and proteins (RNA degradation pathway, ubiquitine and proteasome) also peaked at the same two times (Fig. 7). However, what this study does not address is how these day-night rhythms impact on the level of proteins. This will depend on the life time of the protein, which can vary from minutes to years^86^. If the lifetime of the protein is short, there will be a day-night rhythm in the protein, but if it is longer than 24 h this will not be the case.

### Potential systemic regulators

The master circadian clock in the suprachiasmatic nucleus is entrained to light via the eyes and neuronal circuitry, and peripheral circadian clocks like that in the sinus node are entrained by the master clock and other physiological stimuli. The peripheral clocks are entrained by neurohumoral factors and systemic regulators, including the autonomic nervous system^87^, corticosteroids^87,88^ and possibly thyroid hormone^89^. Presumably the same is true of the sinus node. It is also possible that these same neurohumoral factors and systemic regulators may directly affect pacemaking. It is interesting that transcripts for receptors for catecholamines, ACh, corticosteroids and thyroid hormone all showed day-night rhythms (Figs. 1, 4, S5 and S6). Spoor and Jackson^90^ reported that the heart rate response of isolated atria of the rat (nocturnal like the mouse) to ACh is greater during the day than at night, suggesting that the day-night rhythm in the muscarinic pathway (Fig. S6) has a functional corollary, but perhaps lagging behind mRNA by ~ 12 h. Also Peliciari-Garcia et al.^89^ reported greater triiodothyronine sensitivity (induction of transcript levels) at the end of the night. Therefore, it is possible that the effects of the autonomic nervous system, corticosteroids and thyroid hormone on the sinus node will not just depend on the known day-night rhythms of the autonomic nervous system, corticosteroids and thyroid hormone—they may also depend on the responsiveness of the sinus node to the factors.

### Genes underlying familial and acquired sick sinus syndrome

Familial sick sinus syndrome has been linked to mutations in *Hcn4*^91^, and acquired sick sinus syndrome in ageing^92^, heart failure^93^, atrial fibrillation^94^, diabetes^95^, pulmonary hypertension^96^ and even athletes^11^ has been linked to a downregulation of *Hcn4*. If there is a day-night rhythm in *Hcn4* (P = 0.072), this may impact sick sinus syndrome. For example, athletes have a sinus bradycardia as a result of a downregulation of *Hcn4*^11,78^ and the bradycardia is most marked at night and athletes can have long nocturnal pauses between heart beats at night^14^. Familial sick sinus syndrome has also been linked to mutations in KvLQT1 (*Kcnq1*), Kir2.1 (*Kcnj2*), and calsequestrin 2 (*Casq2*)^27^, all of which show a significant day-night rhythm (*Kcnq1*, *P* = 0.015; *Kcnj2*, *P* = 0.00017; *Ryr2*, *P* = 0.044; *Casq2*, *P* = 0.0080). Once again, the day-night rhythm in the ion channels may impact the phenotype caused by the mutation. Recently, Mesirca et al.^97^ have suggested block of the ACh-activated K^+^ channel to treat sick sinus syndrome. Figure S6 shows that the Kir3.1 subunit of the channel shows a significant day-night rhythm, and this may impact the effect of channel block.

## Conclusion

In conclusion, there is an all-pervasive day-night rhythm in the transcriptome of the pacemaker of the heart, the sinus node. Whether this is responsible for the day-night rhythm in the heart rate or whether it prepares the pacemaker for the demands placed on it during the awake period remains to be determined.

## Methods

Care and use of laboratory animals conformed to the UK Animals (Scientific Procedures) Act 1986 and Directive 2010/63/EU of the European Parliament. Ethical approval for all experimental procedures was granted by the University of Manchester Animal Welfare and Ethical Review body. 12–14 week old adult male C57bl/6j mice were maintained in a 12 h light:12 h dark cycle. The work flow is shown below:

$${\text{Tissue\,harvesting\,at\,ZT}}\,0,\,4,\,8,\,12,\,16\;{\text{and}}\;20\,{\text{h}}\, \to \,{\text{library\,preparation}}\, \to \,{\text{RNAseq}}\, \to \,{\text{Analysis}}$$

Biopsies were collected from the sinus node as we have done previously in many other studies (e.g. Linscheid et al.^98^). We have previously reported the anatomy of the mouse sinus node^99^ and this dictated the position of the biospies. We collected biopsies from the smooth intercaval region between the superior and inferior vena cavae centred on the bifurcation of the sinus node artery. The high expression of sinus node markers (e.g. *Hcn4*) and absence of atrial markers (e.g. atrial natriuretic peptide) have confirmed the nature of the biopsies. Biopsies were collected at six time points at four hourly intervals over the 24 h period: at zeitgeber time (ZT) 0, 4, 8, 12, 16 and 20 h. At each time point, biopsies were collected from three mice. RNA was isolated from the sinus node as described previously^78^. Quantity and integrity of the RNA samples were measured using a 2200 TapeStation (Agilent Technologies) to ensure their suitability. Subsequently, TruSeq Stranded mRNA assays (Illumina) were used in order to produce libraries of more stable, single-stranded cDNA as follows. Total RNA was purified to polyadenylated mRNA via magnetic separation technology, which works through hybridisation of covalent interactions of oligo d(T)_25_ to poly (A) regions present in most eukaryotic mRNA. The mRNA sequences were fragmented into parts via divalent cations at higher temperature, and random primers were used to reverse transcribe the mRNA fragments into single-stranded cDNA. DNA polymerase and RNase H mediated the synthesis of the second cDNA strand produced from RNA oligonucleotides, originating from the 5´ end of the mRNA. The final cDNA library was generated by an addition of a single ‘A’ base, binding of adapters to the fragments and purification and enrichment via a PCR reaction. The cDNA libraries were incorporated into a multiplex system using the adapters; they were then pooled and clustered using a cBlot instrument (Illumina). Optical flow-cells containing the mRNA samples were then paired-end sequenced and mRNA was quantified through repeating 76 cycles twice, using a HiSeq4000 instrument (Illumina). Unmapped paired-end sequences from an Illumina HiSeq4000 sequencer were tested by FastQC (http://www.bioinformatics.babraham.ac.uk/projects/fastqc/). Sequence adapters were removed, and reads were quality trimmed using Trimmomatic_0.36^100^. The reads were mapped against the reference mouse genome (mm10/GRCm38) and counts per gene were calculated using annotation from GENCODE M21 (http://www.gencodegenes.org/) using STAR_2.5.3^101^. Normalisation was carried out using DESeq2_1.18.1^102^. DESeq2 performs an internal normalisation in which a geometric mean is calculated for each gene across all samples. The counts for a gene in each sample is then divided by this mean. The median of these ratios in a sample is the size factor for that sample. This procedure corrects for library size and RNA composition bias, which can arise for example when only a small number of genes are very highly expressed in one experimental condition but not in the other. In figures, the mean ± SEM transcript expression (normalised counts) from the three mice at each time point is plotted. To guide the eye, the data for ZT 0 are also shown for ZT 24 and the data may have been fitted with a sine wave (based on all time points including ZT 24); if fitted, the R^2^ value for the fitted curve is shown in figures. JTK Cycle^24^ was used to test whether a transcript showed a significant day-night rhythm (based on the data at ZT 0, 4, 8, 12, 16 and 20 (but not ZT 24); permutation-based *P* values are shown in figures. In figures, transcripts showing a significant day-night rhythm (permutation-based *P* value < 0.05) are shown in red and are fitted with a sine wave, transcripts showing a trend of a day-night rhythm (permutation-based *P* value > 0.05 but < 0.1) are shown in black and are fitted with a sine wave, and transcripts not showing a significant day-night rhythm (permutation-based *P* value > 0.1) are shown in black and are not fitted with a sine wave. IPA was used to identify potential targets of microRNAs.

## Supplementary Information

Supplementary Information.

Supplementary Data.
