# Supplementary material for: RNAseq shows an all-pervasive day-night rhythm in the transcriptome of the pacemaker of the heart
Source: Sci Rep. 2021 Feb 11;11:3565. doi: 10.1038/s41598-021-82202-7 (PMC7878777; doi:10.1038/s41598-021-82202-7)
Supplement: Supplementary file 1 — Supplementary Information. [file 41598_2021_82202_MOESM1_ESM.pdf]

SUPPLEMENTARY INFORMATION
RNAseq shows an all-pervasive day-night rhythm in the transcriptome of the pacemaker of the heart

Yanwen Wang, Cali Anderson, Halina Dobrzynski, George Hart, Alicia D'Souza and Mark R. Boyett

Supplementary Discussion

Phasing of transcripts

Fig. 7J shows that there was no overall pattern in the transcripts peaking early and late during a 24 h day-night cycle. However, some comments can be made. Regarding the pacemaker *Hcn* transcripts, *Hcn2*, *Hcn3* and *Hcn4* peaked early in the sleep period, whereas *Hcn1* peaked late in the awake period (Fig. 2). This may be important, because the HCN1 channel has faster kinetics and a poorer cAMP sensitivity than the HCN2 and HCN4 isoforms[1]. A switch from the HCN2 and HCN4 isoforms to the HCN1 isoform over 24 h could, therefore, have an impact on pacemaking. Fig. 3 shows that many Ca^{2+} clock transcripts (*Cacna2d1*, *Cacnb2*, *Cacnb4*, *Ryr2*, *Slc8a1*, *Atp2b1*, *Atp2b4*) peaked late in the awake period. Curiously, many adrenergic receptors (*Adrb1*, *Adrb2*, *Adrb3*, *Adra1b*; Figs. 4 and S5) peaked early in the sleep period, whereas the muscarinic receptor peaked late in the awake period (Fig. S6). Many but not all transcripts related to metabolism (involved in the citric acid cycle, acyl-CoA transport, fatty acid β-oxidation and glycolysis) peaked early in the sleep period (Figs. 5 and S12-S14). Could this reflect a build-up of energy reserves in the sleep period?

The significance of the fact that many pathways contain some transcripts peaking in the sleep period and others peaking in the awake period as shown in Fig. 7J is not known. It is likely that this is significant, for example as suggested above in the case of the HCN channels.

Limitations of the study

During analysis of the data using JTK Cycle, the period was set at 24 h. *In vivo*, it is most likely that in an animal under normal 12 h light-12 h dark conditions, the period of a day-night rhythm will be 24 h. There are examples of day-night rhythms with a period of 12 h, but they few in number and more than six measurements over 24 h would be needed to detect them[2]. It is theoretically possible that the period could be longer than 24 h, but no precedents are known and measurements over more than 24 h would be needed to detect them. Only the most abundant transcripts were analysed based on the assumption that poorly expressed transcripts will play little or no role and this assumption may not be strictly correct.

A concern is that the general abundance of a transcript may influence the likelihood of reporting a false negative. To assess this, the adjusted P value was plotted against the average abundance of the transcript over 24 h. Regardless of whether significant transcripts only were considered or non-significant as well as significant transcripts were considered, there was no correlation.

In this study, the transcriptome but not the proteome was studied, and this is an important limitation, because, although 44% of the transcriptome displayed a significant circadian rhythm, the functional consequences of these changes remains an uncertainty until the proteome is measured.

Validation of RNAseq

For RNAseq to be validated, it should be compared to a more accurate technique. RNAseq (1) gives absolute transcript numbers, (2) detects differences in expression of ~10% and (3) has a virtually unlimited dynamic range. Could quantitative PCR (qPCR) be used to validate RNAseq? However, qPCR only gives fold changes with respect to a reference or housekeeper transcript. Therefore, it gives relative rather than absolute expression. Our experience is that with n=8 tissue samples we cannot detect changes as small as 10% using qPCR. qPCR also has a restricted dynamic range. An important weakness of qPCR is the need to normalise data to a reference or housekeeper transcript, which is one that is constant in different samples and conditions; experience shows that this constancy is difficult to assess, prove and obtain. In summary, qPCR is likely to be less accurate than RNAseq and therefore should not be used to validate RNAseq. Another shortcoming of qPCR is that it can only be used for a relatively few pre-identified transcripts (using pre-designed probes); RNAseq in contrast can be used to measure perhaps all or at least a substantial fraction of the entire transcriptome including unknown as well as known transcripts. Therefore, it would be impossible to

validate all of the RNAseq data using qPCR. Despite these arguments, expression of 19 circadian clock transcripts was measured using both RNAseq and qPCR and there was a significant correlation between the two ($P<0.0001$).

Data Supplement files

Supplementary data files are available for download on https://www.nature.com/articles/s41598-021-82202-7. For different functional groups, each file (Excel) shows the transcript, the statistical significance of a day-night rhythm (permutation-based P value calculated using JTK Cycle), the lag time (the zeitgeber time at which expression reaches a peak), and the amplitude of the day-night change (normalised reads or counts). Transcripts showing a significant day-night rhythm are highlighted in yellow. The files relate to Table 1.

HistoneAcetyltransferases.xlsx
CircadianClock.xlsx
TCAcycle.xlsx
EukaryoticInitiationFactors.xlsx
CaMKIIpathway.xlsx
CaClock.xlsx
Glycolysis.xlsx
AutonomicReceptorsPathways.xlsx
MitochondrialSlc25Transporters.xlsx
HistoneDeacetylases.xlsx
TranscriptionFactors.xlsx
FattyAcidBetaOxidation.xlsx
UbiquitinAndProteasome.xlsx
GproteinSubunits.xlsx
AllTranscripts.xlsx
ElectronTransportChain.xlsx
RNApolymerases.xlsx
SoluteSlcCarriers.xlsx
CalciumIonTransport.xlsx
GproteinCoupledReceptors.xlsx
ExtracellularMatrix.xlsx
IonChannelSubunits.xlsx
GapJunctionSubunits.xlsx

Enriched pathways peaking in the sleep and awake periods (Fig. 7J) are given in the file below; data were analysed through the use of IPA (QIAGEN Inc., https://www.qiagenbioinformatics.com/products/ingenuitypathway-analysis)[3].

Enriched pathways at ZT 2-8 and ZT 16-18h.xlsx

References

1. Altomare C, Terragni B, Brioschi C, Milanesi R, Pagliuca C, Viscomi C, Moroni A, Baruscotti M, DiFrancesco D. Heteromeric HCN1-HCN4 channels: a comparison with native pacemaker channels from the rabbit sinoatrial node. *Journal of Physiology*. 2003;549:347-359.
2. Thosar SS, Butler MP, Shea SA. Role of the circadian system in cardiovascular disease. *Journal of Clinical Investigation*. 2018;128:2157-2167.
3. Krämer A, Green J, Pollard J, Jr., Tugendreich S. Causal analysis approaches in Ingenuity Pathway Analysis. *Bioinformatics*. 2014;30:523-30.
4. Eppinga RN, Hagemeijer Y, Burgess S, Hinds DA, Stefansson K, Gudbjartsson DF, van Veldhuisen DJ, Munroe PB, Verweij N, van der Harst P. Identification of genomic loci associated with resting heart rate and shared genetic predictors with all-cause mortality. *Nature Genetics*. 2016;48:1557-1563.
5. Mezzavilla M, Iorio A, Bobbo M, D'Eustacchio A, Merlo M, Gasparini P, Ulivi S, Sinagra G. Insight into genetic determinants of resting heart rate. *Gene*. 2014;545:170-174.
6. Ramírez J, Duijvenboden Sv, Ntalla I, Mifsud B, Warren HR, Tzanis E, Orini M, Tinker A, Lambiase PD, Munroe PB. Thirty loci identified for heart rate response to exercise and recovery implicate autonomic nervous system. *Nature Communications*. 2018;9:1947.
7. Nagy R, Boutin TS, Marten J, Huffman JE, Kerr SM, Campbell A, Evenden L, Gibson J, Amador C, Howard DM, Navarro P, Morris A, Deary IJ, Hocking LJ, Padmanabhan S, Smith BH, Joshi P, Wilson JF, Hastie ND, Wright AF, McIntosh AM, Porteous DJ, Haley CS, Vitart

V, Hayward C. Exploration of haplotype research consortium imputation for genome-wide association studies in 20,032 Generation Scotland participants. *Genome Medicine*. 2017;9:23.
8. Eijgelsheim M, Newton-Cheh C, Sotoodehnia N, de Bakker PIW, Müller M, Morrison AC, Smith AV, Isaacs A, Sanna S, Dörr M, Navarro P, Fuchsberger C, Nolte IM, de Geus EJC, Estrada K, Hwang S-J, Bis JC, Rückert I-M, Alonso A, Launer LJ, Hottenga JJ, Rivadeneira F, Noseworthy PA, Rice KM, Perz S, Arking DE, Spector TD, Kors JA, Aulchenko YS, Tarasov KV, Homuth G, Wild SH, Marroni F, Gieger C, Licht CM, Prineas RJ, Hofman A, Rotter JI, Hicks AA, Ernst F, Najjar SS, Wright AF, Peters A, Fox ER, Oostra BA, Kroemer HK, Couper D, Völzke H, Campbell H, Meitinger T, Uda M, Witteman JCM, Psaty BM, Wichmann HE, Harris TB, Kääb S, Siscovick DS, Jamshidi Y, Uitterlinden AG, Folsom AR, Larson MG, Wilson JF, Penninx BW, Snieder H, Pramstaller PP, van Duijn CM, Lakatta EG, Felix SB, Gudnason V, Pfeufer A, Heckbert SR, Stricker BHC, Boerwinkle E, O'Donnell CJ. Genome-wide association analysis identifies multiple loci related to resting heart rate. *Human Molecular Genetics*. 2010;19:3885-3894.
9. Reed GJ, Boczek NJ, Etheridge SP, Ackerman MJ. CALM3 mutation associated with long QT syndrome. *Heart Rhythm*. 2015;12:419-422.
10. Cheung JY, Zhang X-Q, Song J, Gao E, Rabinowitz JE, Chan TO, Wang J. Phospholemman: a novel cardiac stress protein. *Clinical and Translational Science*. 2010;3:189-196.
11. Kreusser MM, Backs J. Integrated mechanisms of CaMKII-dependent ventricular remodeling. *Frontiers in Pharmacology*. 2014;5:36.
12. Rose BA, Force T, Wang Y. Mitogen-activated protein kinase signaling in the heart: angels versus demons in a heart-breaking tale. *Physiological Reviews*. 2010;90:1507-46.
13. Redwood CS, Moolman-Smook JC, Watkins H. Properties of mutant contractile proteins that cause hypertrophic cardiomyopathy. *Cardiovascular Research*. 1999;44:20-36.

Table S1. Day-night rhythm in ion channels. The table shows the ion channel transcript, the statistical significance of a day-night rhythm (permutation-based P value calculated using JTK Cycle), the lag time (the zeitgeber time at which expression reaches a peak), the average expression (normalised reads or counts) over 24 h (based on measurements at six time points over 24 h and n=3 mice at each time point), the amplitude of the day-night change (normalised reads or counts), and the amplitude of the day-night change as a percentage of the average expression. The amplitude is the change in the transcript from the mean and, therefore, the total change from day to night is 2×amplitude. Ion channel subunits showing a significant day-night rhythm are highlighted.

Ion channel subunit	Permutation-based P value	Lag time (h)	Average (normalised reads)	Amplitude (normalised reads)	Amplitude/average (%)
Chrnb4	3.71E-05	6	320.997755	153.35743	47.7752344
Trpm7	0.000103647	16	852.932788	506.002526	59.3250175
Cacnb1	0.000103647	4	429.403206	125.163346	29.1482094
Kcnj2	0.000167488	20	559.648992	245.70297	43.9030489
Kcnk3	0.000265156	4	11277.4541	3258.2501	28.8917168
Kcng2	0.000942737	2	3520.29257	741.476027	21.0629092
Itpr1	0.000942737	18	1425.4757	447.01348	31.3588986
Clcn3	0.000942737	16	821.166884	316.038966	38.4865698
Kcna6	0.000942737	6	485.811463	173.846183	35.7847018
Chrnb2	0.000942737	8	71.6064121	32.9988581	46.0836636
Gja5	0.001392373	6	2266.78102	1221.10367	53.8695027
Pkd2	0.001392373	18	1275.69728	618.749178	48.5028218
Grik5	0.001392373	6	697.521165	133.994613	19.2101143
Gria3	0.001392373	18	193.460124	116.574635	60.2577071
Trpv2	0.001392373	6	260.946812	49.6414583	19.0235925
Kcnc4	0.001392373	6	110.763551	45.2672065	40.8683237
Ano10	0.002026164	6	1749.39937	375.938069	21.489551
Gabra3	0.002026164	18	416.270824	143.173727	34.39437
Ano4	0.002026164	18	285.104374	120.305716	42.1970781
Clic5	0.002907354	18	4083.14028	1985.71477	48.6320487
Clcn2	0.002907354	12	219.407273	52.4328889	23.8975163
Kcns3	0.002907354	8	78.7105044	38.6571781	49.1131118
Clcn4	0.004116638	18	2497.8088	736.41614	29.4824864
Gabre	0.004116638	16	222.248774	115.83378	52.1189734
Itpr2	0.004116638	16	630.655508	107.113168	16.984418
Trpc1	0.004116638	18	166.661756	80.3128043	48.1891024
Cacng7	0.004116638	6	294.501049	45.3585519	15.4018303
Kcnj3	0.005755688	16	6604.58281	2930.18506	44.3659372
Cacnb2	0.005755688	18	1366.3809	552.468936	40.4330106
Chrnb1	0.005755688	18	260.393722	66.9497287	25.7109611
Clcnkb	0.005755688	20	17.8579937	4.55825545	25.5250144
Cacna2d1	0.007951044	18	1119.34669	584.184209	52.1897473
Cacnb4	0.007951044	18	44.9406593	24.7954268	55.173705
Asic2	0.007951044	6	28.6033305	14.3923462	50.3170293
Cftr	0.007951044	14	10.740125	5.86228713	54.583044

Table S1 continued.

Ion channel subunit	Permutation-based P value	Lag time (h)	Average (normalised reads)	Amplitude (normalised reads)	Amplitude/average (%)
Clic1	0.010858335	4	3010.36926	693.983354	23.0530973
Ano1	0.010858335	12	583.676612	202.293225	34.6584429
Kcnd3	0.010858335	16	403.795299	167.09024	41.379937
Lrrc8d	0.010858335	6	517.566232	72.0127483	13.9137262
Clcn5	0.010858335	18	232.462621	61.1671921	26.3127
Htr3b	0.010858335	4	16.2089256	12.8053266	79.0016994
Scn2a	0.010858335	14	32.6497068	7.18674258	22.0116604
Kcnq1	0.014666774	4	2756.47668	447.138375	16.2213734
Hcn2	0.014666774	6	684.693368	203.239941	29.6833518
Mcoln1	0.014666774	8	1251.47876	124.841839	9.97554602
Catsper2	0.014666774	16	96.1213706	23.39259	24.3365131
Hvcn1	0.019603809	8	276.529108	111.277409	40.2407578
Kcnq4	0.019603809	8	239.683399	41.3112389	17.2357531
Gabrq	0.025939829	16	49.9903126	35.9389557	71.8918403
Aqp11	0.025939829	8	80.9381681	20.5077446	25.3375448
Kcng4	0.025939829	6	20.3539519	14.8891773	73.1512848
Tpcn1	0.033992784	6	3262.56803	546.104855	16.7384971
Hcn1	0.033992784	18	892.285632	335.951501	37.650668
Grin3a	0.033992784	0	50.2483746	16.381666	32.6013849
Scn11a	0.033992784	6	13.723683	7.46721986	54.4111945
Ryr2	0.044132525	18	19459.0532	11602.1488	59.6233985
Aqp7	0.044132525	6	737.956836	324.97834	44.037581
P2rx4	0.044132525	8	1074.18249	142.260159	13.2435746
Ano8	0.044132525	6	797.878472	109.328735	13.7024295
Kcnt2	0.044132525	18	121.281086	72.3219064	59.6316446
Asic1	0.044132525	4	118.855961	42.9155281	36.1071736
P2rx7	0.044132525	10	349.75942	38.3437142	10.9628825
Trpm3	0.044132525	18	55.8486287	20.3649196	36.4644936
Cngb1	0.044132525	12	14.0474485	4.6673315	33.2254751
Ryr3	0.056784681	18	845.38585	398.979562	47.1949656
Cacnb3	0.056784681	6	543.739049	126.479676	23.2610986
P2rx6	0.056784681	8	288.902512	48.3467023	16.7346078
Kcnq2	0.056784681	6	85.3520184	45.5947391	53.4196378
Grin1	0.056784681	6	39.7141108	26.1953568	65.9598221
Kcnn4	0.056784681	6	116.577635	24.1952112	20.7545909
Cacna1i	0.056784681	8	35.1671637	17.9833595	51.1367924
Hcn4	0.072433859	4	6209.67453	1082.61717	17.4343625
Kcnj8	0.072433859	4	1105.26809	369.399847	33.4217418
Itpr3	0.072433859	10	645.673555	97.4861608	15.0983667
Gjc1	0.072433859	20	487.621507	57.4608828	11.7839107
Kcnk5	0.072433859	8	120.524629	20.7399551	17.2080638

Table S1 continued.

Ion channel subunit	Permutation-based P value	Lag time (h)	Average (normalised reads)	Amplitude (normalised reads)	Amplitude/average (%)
Cngb3	0.072433859	18	18.9086894	9.02480604	47.728353
P2rx1	0.072433859	6	22.0101819	6.7455155	30.6472501
Gja1	0.091625931	18	5730.61422	808.01373	14.0999498
Lrrc8c	0.091625931	20	1128.98104	144.034749	12.7579423
Chrna3	0.091625931	6	178.743862	94.3173772	52.7667782
Kcnd2	0.091625931	16	186.851001	71.1560635	38.0817138
Kcnh6	0.091625931	4	36.9189438	8.32876524	22.5595978
Chrna2	0.091625931	12	34.0616854	8.16032311	23.9574848
P2rx2	0.091625931	6	16.5936113	8.01746226	48.3165607
Kcnq5	0.091625931	8	11.1749507	7.9562229	71.1969393
Trpv1	0.091625931	8	19.8104268	7.93845843	40.0721222
Ano3	0.091625931	18	18.7785957	7.68352044	40.9163739
Asic3	0.091625931	12	10.4954123	3.60430182	34.3416886
Vdac2	0.114969165	2	12331.1939	1128.33467	9.15024675
Vdac3	0.114969165	2	7155.57545	802.304589	11.2123
Gjd3	0.114969165	2	166.281511	36.0986606	21.7093653
Hcn3	0.114969165	6	30.9507713	21.1455435	68.3199243
Gjc2	0.114969165	4	67.2022318	20.6613075	30.7449723
Kcnh3	0.114969165	8	13.1419895	3.75853285	28.5994205
P2rx3	0.114969165	10	10.4626129	2.23732456	21.3839945
Kcnh2	0.143133982	6	6437.9481	662.210543	10.2860497
Cacna1d	0.143133982	16	796.983817	195.300633	24.5049685
Aqp4	0.143133982	14	73.602721	27.7462302	37.6972887
Trpc3	0.143133982	20	138.453222	24.4192184	17.6371616
Kcnj15	0.143133982	18	110.978682	16.764589	15.1061345
Kcnj10	0.143133982	8	61.6340721	13.3392386	21.6426371
Trpc4	0.143133982	20	17.6065427	2.69827659	15.3254198
Aqp5	0.176851105	14	56.9714514	26.8126787	47.0633591
Ryr1	0.176851105	8	33.9118918	12.1987972	35.9720339
Clic4	0.264142705	20	13858.0979	2430.49334	17.5384339
Vdac1	0.264142705	2	23942.5097	2169.16116	9.05987378
Cacna1c	0.264142705	18	2396.09533	987.41277	41.209244
Scn3b	0.264142705	8	205.123601	69.672383	33.966049
Gria1	0.264142705	18	256.932081	56.7590961	22.0910896
Kcnf1	0.264142705	22	67.2030078	6.71929104	9.99849749
Ano5	0.264142705	20	23.0999282	4.97075596	21.518491
Kcnc1	0.264142705	6	13.0219436	3.78807733	29.0899533
Kcnj12	0.31943719	22	748.792151	100.401218	13.4084229
Gja4	0.31943719	2	578.587147	68.3790458	11.8182794
Kcnn1	0.31943719	2	507.382182	54.4276306	10.7271466
Gjc3	0.31943719	12	57.7319269	21.8902373	37.9170391

Table S1 continued.

Ion channel subunit	Permutation-based P value	Lag time (h)	Average (normalised reads)	Amplitude (normalised reads)	Amplitude/average (%)
Gabrr2	0.31943719	22	44.1876835	14.0325949	31.7568015
Cacna1b	0.31943719	6	38.7646713	12.0859045	31.1776266
Grid2	0.31943719	20	19.0837803	4.47336906	23.4406862
Cacna1g	0.383706374	2	2045.45497	196.873574	9.62492829
Kcnn2	0.383706374	18	500.430213	131.225722	26.2225818
Kcnv2	0.383706374	16	188.7664	74.4915041	39.4622688
Trpm6	0.383706374	14	22.7590724	6.96443479	30.600697
Aqp1	0.457886631	22	20524.5977	1170.52535	5.70303675
Cacna2d3	0.457886631	18	509.973019	67.5280979	13.241504
Lrrc8b	0.457886631	16	198.660302	48.646344	24.4871992
Tpcn2	0.457886631	4	313.918926	20.0651656	6.39183049
Trpm2	0.457886631	10	33.4908134	11.4161806	34.0874987
Best1	0.457886631	18	43.8842947	7.7055992	17.5588995
Kcnd1	0.457886631	4	77.4848784	7.67252839	9.90196868
Scn3a	0.457886631	14	26.4101501	4.34904987	16.4673425
Mcoln3	0.457886631	16	16.5825514	1.93011671	11.6394436
Trpm4	0.54292154	6	1010.03007	107.412946	10.6346285
Clcn6	0.54292154	4	729.01663	64.3620397	8.82861064
P2rx5	0.54292154	0	806.932865	62.5326797	7.74942779
Trpv4	0.54292154	22	350.167259	49.2215341	14.0565781
Kcnh1	0.54292154	6	30.759413	4.79844601	15.5999271
Grin3b	0.54292154	6	10.9549911	4.13407391	37.7368989
Gjb4	0.54292154	18	11.0652114	4.09265507	36.9866867
Gria4	0.639745804	18	50.1908065	19.7797125	39.4090351
Kcnn3	0.639745804	16	60.99426	9.7177689	15.9322679
Kcnq3	0.639745804	6	26.6753457	7.0765448	26.5284089
Kcnk1	0.639745804	14	22.4385464	6.68241057	29.7809424
Kcnk2	0.639745804	12	41.6979868	4.33203076	10.3890645
Cacna1h	0.749267486	10	7326.58411	867.097188	11.8349448
Kcnj11	0.749267486	4	4204.7991	335.973893	7.9902484
Clcn1	0.749267486	16	546.914641	86.3647043	15.7912584
Gja3	0.749267486	0	625.132558	75.3682303	12.0563598
Ano6	0.749267486	18	1420.06673	72.0210559	5.07166703
Best3	0.749267486	20	139.163866	49.2177886	35.3667873
Nalcn	0.749267486	14	68.673427	11.638228	16.9472072
Kcnc2	0.749267486	18	43.6528761	8.48104433	19.4283747
Kcna2	0.749267486	16	365.442679	8.17725548	2.23763013
Clic6	0.749267486	14	110.475663	7.51614309	6.80343789
Kcng1	0.749267486	14	11.1410934	2.33768194	20.9825182
Lrrc8a	0.872348835	0	1022.14626	178.917348	17.5040848
Scn2b	0.872348835	6	142.580574	22.3122321	15.6488584

Table S1 continued.

Ion channel subunit	Permutation-based P value	Lag time (h)	Average (normalised reads)	Amplitude (normalised reads)	Amplitude/average (%)
Cacna1e	0.872348835	12	23.4307286	4.9329317	21.0532578
Gjb2	0.872348835	10	11.8248421	1.71321989	14.4883109
Scn5a	1	18	7499.60525	885.089568	11.8018154
Kcnj5	1	18	10446.2695	870.15038	8.32977149
Scn1b	1	10	1821.10032	211.071512	11.5903286
Scn4b	1	10	1290.91676	197.814206	15.3235447
Scn4a	1	10	722.612772	145.232402	20.0982334
Cacna2d2	1	12	4807.1002	144.791814	3.01204069
Kcnb1	1	18	1502.30722	80.3255725	5.34681398
Kcna5	1	0	625.899018	62.7421168	10.0243194
Clcn7	1	0	1612.02752	53.8511177	3.34058303
Kcnk6	1	18	264.037362	48.7519754	18.4640443
Pkd2l2	1	18	355.403822	35.2662645	9.92287149
Cacna1a	1	18	326.887238	32.5289693	9.95112854
Scn10a	1	10	178.614772	29.8497948	16.7118287
Gjb5	1	22	117.698185	28.5640146	24.2688658
Kcna1	1	12	137.986886	15.6424071	11.3361548
Grin2c	1	0	94.9232225	14.6001722	15.381033
Kcnc3	1	0	81.2956026	14.1100696	17.356498
Grin2d	1	10	129.41409	13.606023	10.5135561
Kcnma1	1	10	29.2200565	13.1006905	44.834583
Kcnk13	1	0	80.5261714	12.2630791	15.2286876
Grik3	1	10	79.6236299	10.9698043	13.7770714
Kcna7	1	18	74.398852	10.5646343	14.1999965
Scn9a	1	10	11.8778657	8.21894096	69.1954359
Mcoln2	1	10	30.5209625	7.37311549	24.1575458
Kcna4	1	18	69.9235649	7.11611795	10.1769954
Scnn1a	1	12	60.3674679	6.86225734	11.3674759
Kcnj14	1	0	172.846081	6.80042346	3.93438105
Cacna1s	1	6	117.070921	6.54627	5.5917131
Glrb	1	10	27.7666501	6.46617228	23.2875491
Clic3	1	22	62.6083043	5.72831047	9.14944198
Gjb3	1	0	20.6566133	5.32529629	25.7801035
Kcnt1	1	18	77.9412926	4.13760685	5.30861975
Gabrg3	1	0	50.3188809	3.50515048	6.96587527
Gabrb3	1	8	15.2836776	3.28408494	21.4875308
Trpc6	1	10	11.040494	3.03563552	27.4954682
Gabrr1	1	18	24.5149091	2.48364035	10.1311424
Scn8a	1	12	14.5539555	1.69617712	11.6544064
Grik2	1	18	18.3558945	1.61875136	8.81870051
Cnga3	1	10	10.2734949	1.51395115	14.7364765

Table S2. Day-night rhythm in transcripts related to the immune system. The table lists the transcripts and the permutation-based P values for a significant day-night rhythm in the transcripts.

Transcript	Permutation-based P value
Bmp3	0.00411664
Bmp6	0.01960381
Ccr2	0.01960381
Cd163	0.01466677
Cd1d1	0.00202616
Cd3g	0.04413253
Cklf	0.01466677
Cmtm3	0.00575569
Cmtm5	0.02593983
Ctf1	0.00575569
Cx3cl1	0.01960381
Cybb	0.00041183
Fgf2	0.00062833
Foxp3	0.02593983
Gdf5	0.03399278
Gpi1	0.00026516
Grn	0.01960381
Hmgb1	0.00016749
Il13ra1	0.00795104
Il1rap	0.00795104
Il33	0.00062833
Il7	0.00026516
Ildr2	0.03399278

Transcript	Permutation-based P value
Inhba	0.03399278
Kitl	0.00041183
Lif	0.00094274
Lta	0.00170927
Mif	0.00795104
Mmp9	0.01466677
Mrc1	0.03399278
Msmp	0.00795104
Nampt	0.00010365
Osmr	0.00575569
Ptprc	0.01085834
Rora	0.00139237
Slurp1	0.01085834
Tap2	0.00575569
Thy1	0.0000627
Tnfrsf12a	0.01466677
Tnfsf13b	0.01085834
Vegfa	0.01466677
Wnt2	0.03399278
Wnt2b	0.03399278
Wnt5b	0.04413253
Wnt9b	0.02593983

Table S3. GWAS-identified genes related to resting heart rate showing a significant day-night rhythm or a trend of one. The table lists the genes, the permutation-based P values for a significant day-night rhythm in the genes, the GWAS source identifying the genes, and notes concerning the function of the genes.

Gene	Permutation-based P value	Source	Notes
Met	1.19E-05	Eppinga *et al.*[4]	Receptor tyrosine kinase; PI3K is a downstream target, which is known to regulate HCN4
Lzic	0.00017	Eppinga *et al.*[4]	
Alg10	0.00027	Eppinga *et al.*[4]	Alg10b reported; modulates ether-a-go-go (EAG) K^+ channel
Mkln1	0.00041	Eppinga *et al.*[4]	Component of CTLH E3 ubiquitin-protein ligase complex
Klhl42	0.00041	Eppinga *et al.*[4]	Ubiquitin-protein transferase activity
Gmppb	0.00094	Eppinga *et al.*[4]	
Adck1	0.00094	Eppinga *et al.*[4]	
Canx	0.0014	Mezzavilla *et al.*[5]	Calnexin; assists protein folding and quality control in the endoplasmic reticulum
Capza2	0.0014	Ramirez *et al.*[6]	
Ccdc141	0.0020	Eppinga *et al.*[4]	
Asph	0.0020	Nagy *et al.*[7]	
Slc12a9	0.0020	Eijgelsheim *et al.*[8]	Cation-chloride cotransporter 6 (CCC6) or cation-chloride cotransporter-interacting protein 1 (CIP1)
Prkar2a	0.0029	Eppinga *et al.*[4]	cAMP-dependent protein kinase type II-alpha regulatory subunit
Ppargc1a	0.0029	Eppinga *et al.*[4]	Transcriptional coactivator that regulates energy metabolism genes
Tbx20	0.0029	Eppinga *et al.*[4]	Transcription factor
Ephb4	0.0029	Eppinga *et al.*[4]	Receptor tyrosine kinase
Tanc1	0.0029	Eppinga *et al.*[4]	
Rnf220	0.0041	Eppinga *et al.*[4]	E3 ubiquitin-protein ligase RNF220
Calcrl	0.0041	Eppinga *et al.*[4]	Receptor for calcitonin gene-related peptide
Chrm2	0.0041	Eppinga *et al.*[4]	Muscarinic M2 receptor
Arhgap10	0.0058	Eppinga *et al.*[4]	
Ppil1	0.0058	Eppinga *et al.*[4]	Protein folding
Ttn	0.0080	Eppinga *et al.*[4]	Titin
Fen1	0.0080	Eppinga *et al.*[4]	
Fam227a	0.0080	Eppinga *et al.*[4]	
Ddx17	0.0080	Eppinga *et al.*[4]	Implicated in translation initiation, nuclear and mitochondrial splicing, and ribosome and spliceosome assembly
Gpatch2	0.011	Eppinga *et al.*[4]	
Qrich1	0.011	Eppinga *et al.*[4]	
Frmd4b	0.011	Eppinga *et al.*[4]	
Srrt	0.011	Eppinga *et al.*[4]	Plays role in RNA-mediated gene silencing by miRNAs
Ache	0.011	Eppinga *et al.*[4]	Acetylcholine esterase
Map3k10	0.011	Eppinga *et al.*[4]	Mitogen-activated protein kinase kinase kinase 10
Cpne8	0.011	Ramirez *et al.*[6]	
Srebf1	0.011	Eppinga *et al.*[4]	Transcription factor

Table S3 continued.

Gene	Permutation-based P value	Source	Notes
Ufsp1	0.015	Eppinga *et al.*[4]	Protease
Pcolce	0.015	Eppinga *et al.*[4]	
Arhgef40	0.020	Eppinga *et al.*[4]	
Flrt2	0.020	Eppinga *et al.*[4]	
Apoc1	0.026	Eppinga *et al.*[4]	
Cdh11	0.034	Eppinga *et al.*[4]	Cadherin-11
Apoe	0.034	Eppinga *et al.*[4]	
Tomm22	0.034	Eppinga *et al.*[4]	
Dsp	0.034	Eppinga *et al.*[4]	Desmoplakin
Cd46	0.044	Eppinga *et al.*[4]	
Ip6k1	0.044	Eppinga *et al.*[4]	Inositol hexakisphosphate kinase 1; link to AMP kinase
Lamb2	0.057	Eppinga *et al.*[4]	
Cby1	0.057	Eppinga *et al.*[4]	Interacts with beta-catenin, inhibiting beta-catenin-mediated transcriptional activation
Hcn4	0.072	Eppinga *et al.*[4]	HCN4
Des	0.092	Eppinga *et al.*[4]	Desmin
Cdc23	0.092	Eppinga *et al.*[4]	Ubiquitin-protein transferase activity
Gja1	0.092	Eppinga *et al.*[4]	Cx43
Gng11	0.092	Eppinga *et al.*[4]	G protein subunit gamma 11
Ndrg2	0.092	Eppinga *et al.*[4]	
Gtpbp1	0.092	Eppinga *et al.*[4]	Promotes degradation of target mRNA species and plays a role in the regulation of circadian mRNA stability

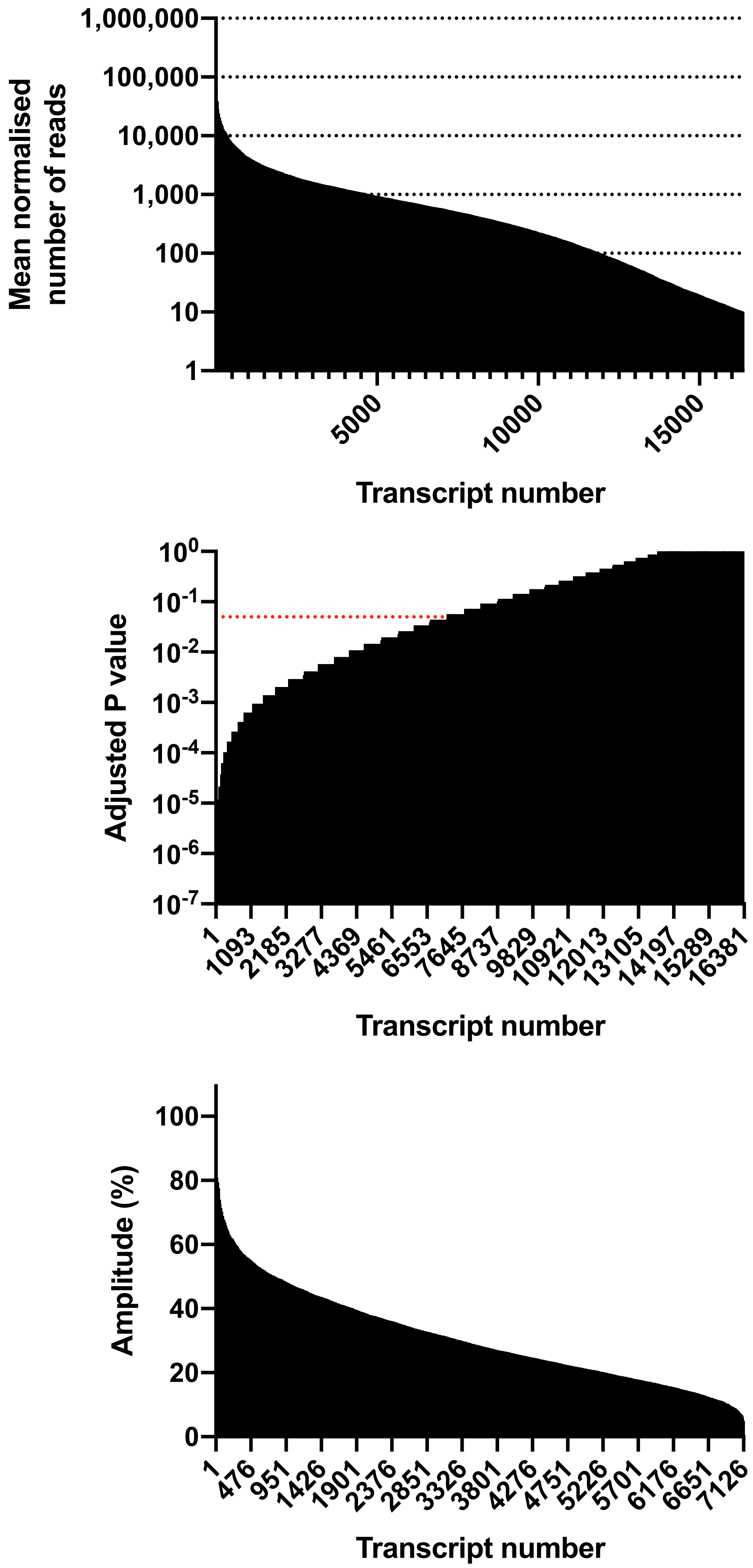

Fig. S1. Transcriptome showing a day-night rhythm. A, average expression (normalised reads or counts) over 24 h of the 16,387 most abundant transcripts. **B**, permutation-based P value from JTK Cycle for a significant day-night rhythm for the 16,387 transcripts; P=0.05 is shown by a red dotted line. **C**, amplitude of the day-night rhythm (expressed as a percentage of the average expression over 24 h) for the 7,134 transcripts showing a significant day-night rhythm. In each panel, transcripts are ranked in terms of the variable shown.

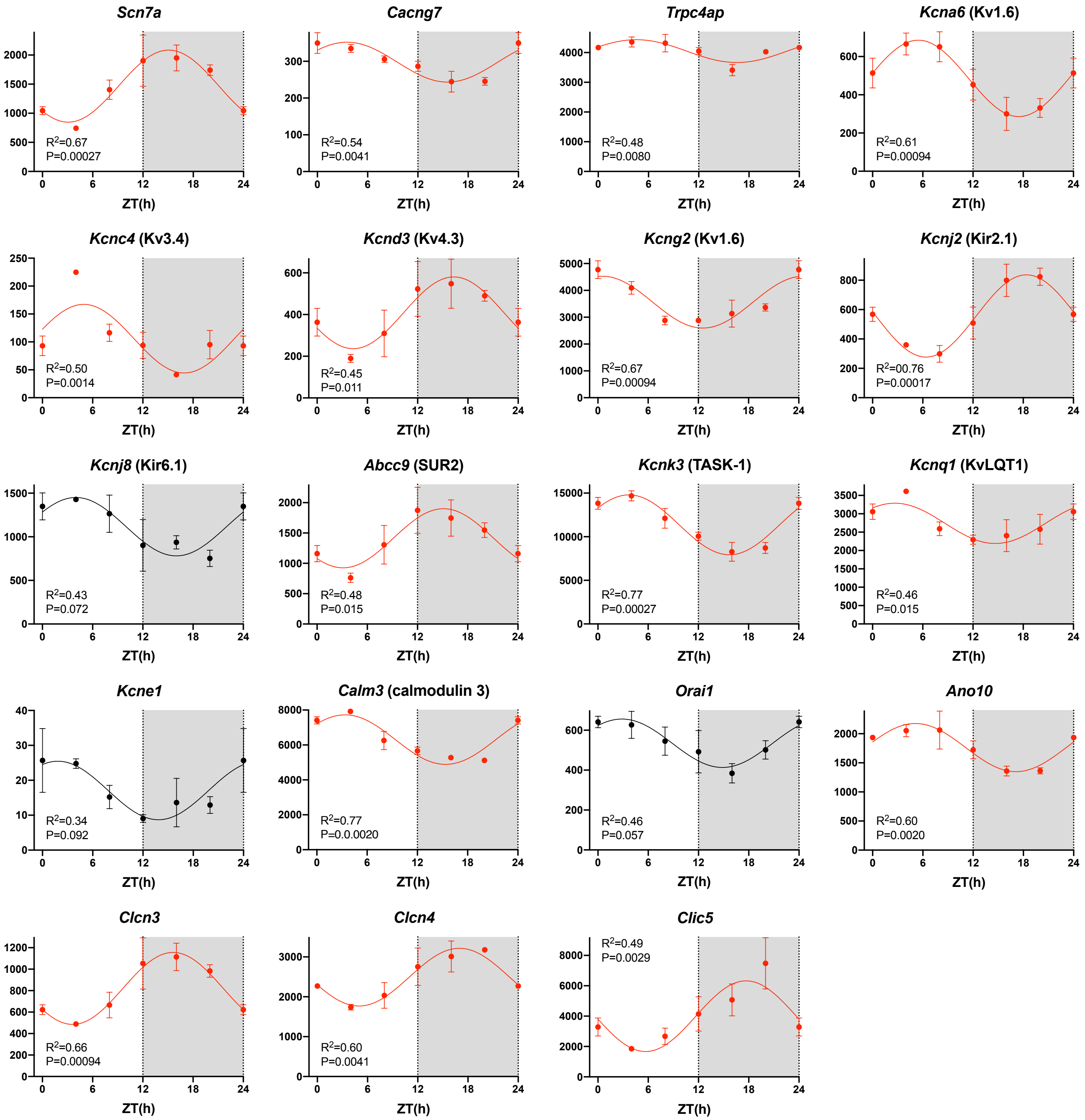

Fig. S2. Ion channels subunits. Abundance (normalised counts) of transcripts for ion channel subunits is shown over 24 h. Calm3 is a regulator of Kcnq1[9]. Gene name and common name (in parenthesis) given; mean ± SEM transcript abundance shown (n=3) at six ZT time points over 24 h (24 h data repeat of 0 h data); significant data (P<0.05) or data showing a trend towards significance (0.05<P<0.1) have been fitted with a sine wave by a least squares fitting method and the R^2 value is given; the permutation-based P value from JTK Cycle for significance of a day-night rhythm is given; significant data are shown in red and non-significant data are shown in black.

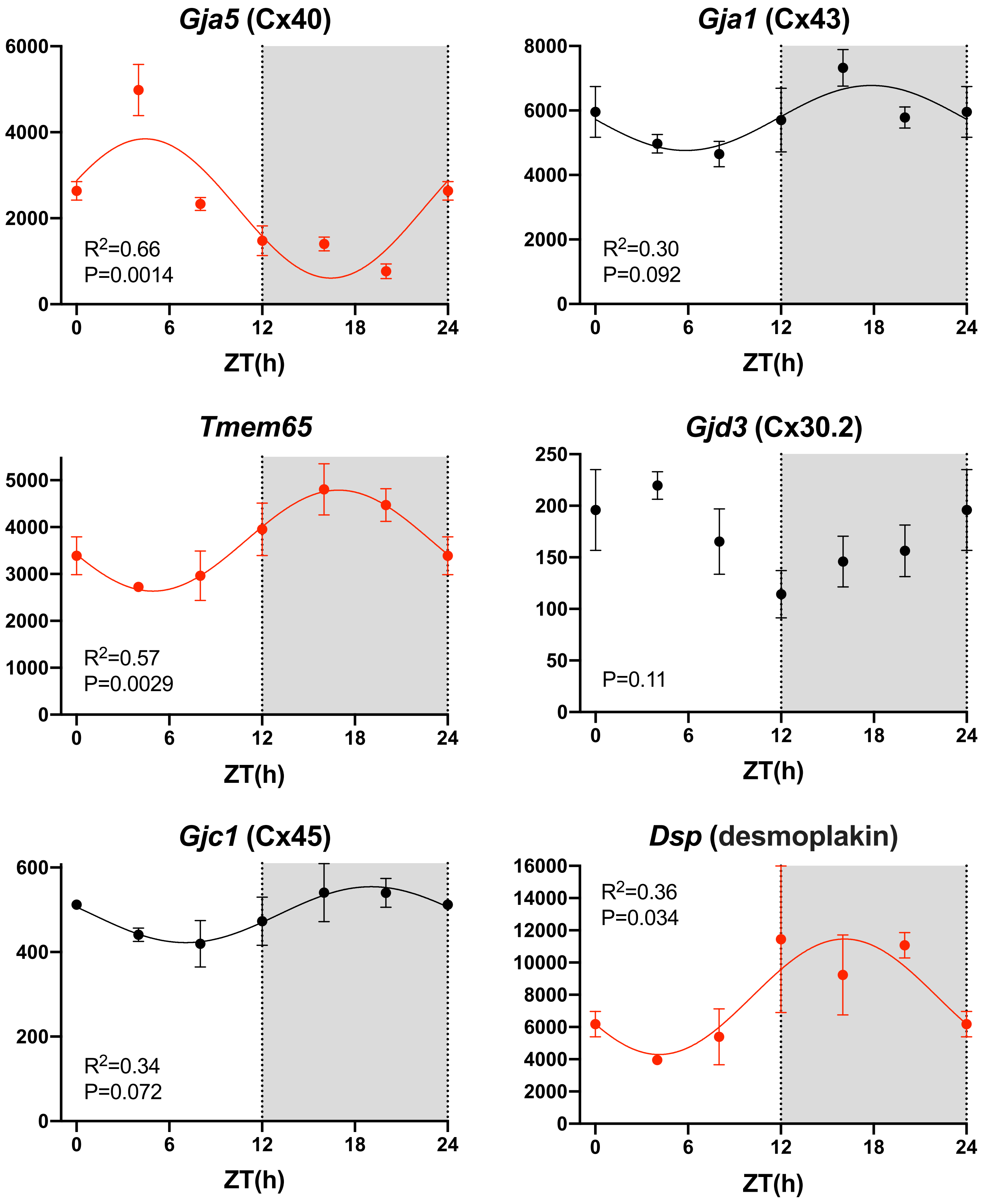

Fig. S3. Cell-cell coupling. Abundance (normalised counts) of gap junction and desmosome transcripts is shown over 24 h. Gene name and common name (in parenthesis) given; mean ± SEM transcript abundance shown (n=3) at six ZT time points over 24 h (24 h data repeat of 0 h data); significant data ($P<0.05$) or data showing a trend towards significance ($0.05<P<0.1$) have been fitted with a sine wave by a least squares fitting method and the R^2 value is given; the permutation-based P value from JTK Cycle for significance of a day-night rhythm is given; significant data are shown in red and non-significant data are shown in black.

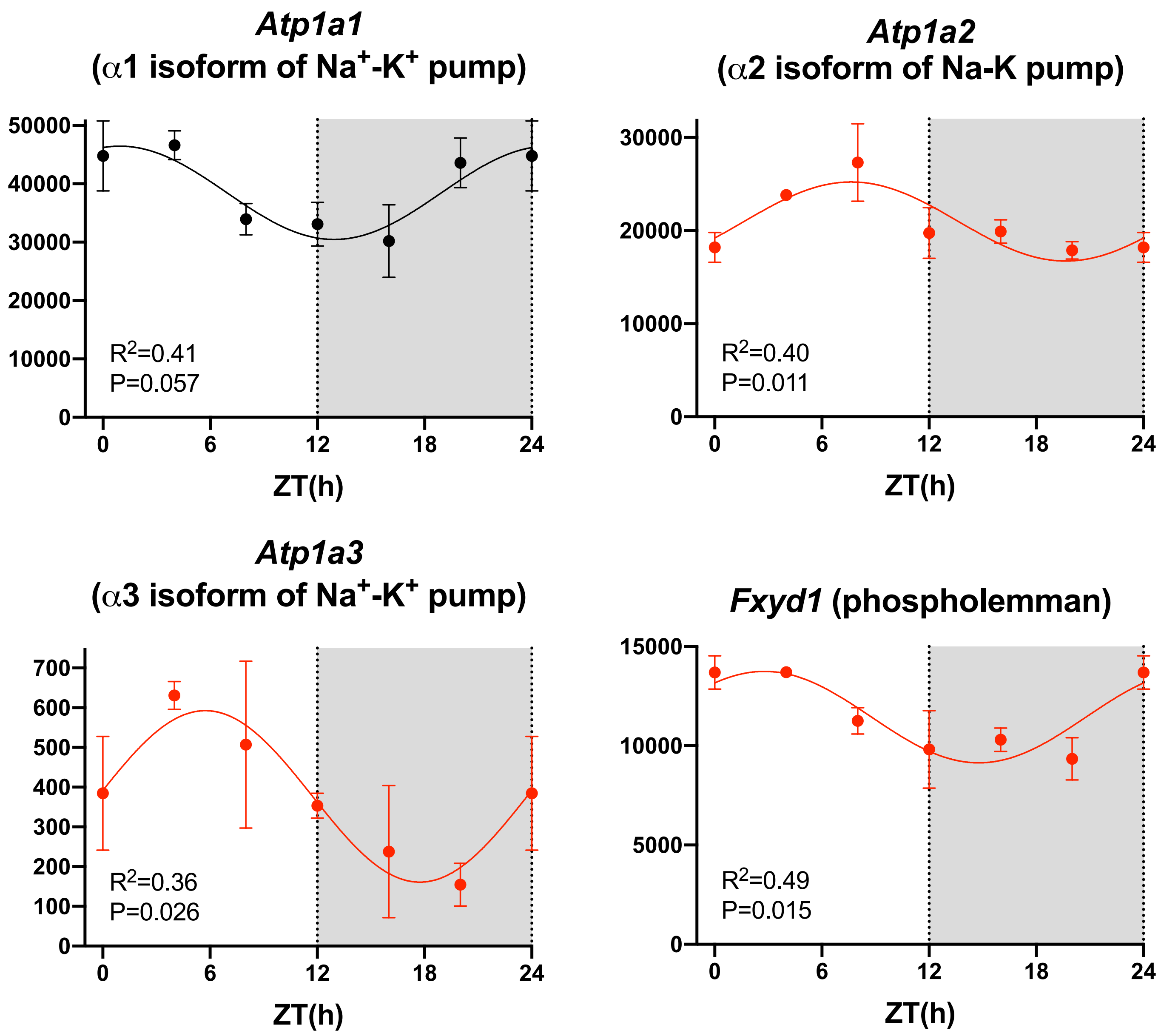

Fig. S4. Na^+-K^+ pump subunits. Abundance (normalised counts) of transcripts for Na^+-K^+ pump subunits and phospholemman, a regulator of the Na^+-K^+ pump[10], is shown over 24 h. Gene name and common name (in parenthesis) given; mean ± SEM transcript abundance shown (n=3) at six ZT time points over 24 h (24 h data repeat of 0 h data); significant data ($P<0.05$) or data showing a trend towards significance ($0.05<P<0.1$) have been fitted with a sine wave by a least squares fitting method and the R^2 value is given; the permutation-based P value from JTK Cycle for significance of a day-night rhythm is given; significant data are shown in red and non-significant data are shown in black.

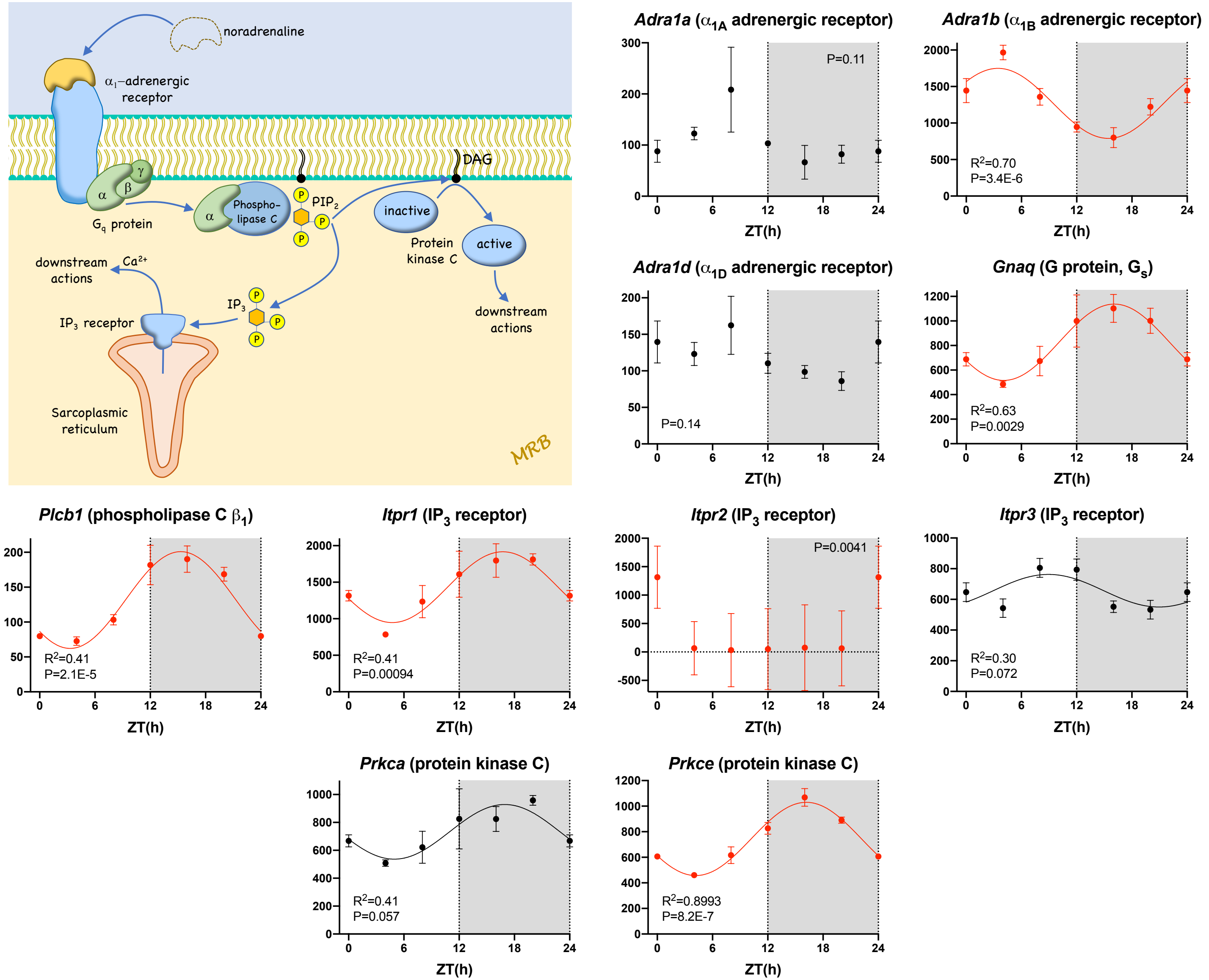

Fig. S5. α-adrenergic receptor pathway. Abundance (normalised counts) of α-adrenergic receptor pathway transcripts is shown over 24 h. Gene name and common name (in parenthesis) given; mean ± SEM transcript abundance shown (n=3) at six ZT time points over 24 h (24 h data repeat of 0 h data); significant data (P<0.05) or data showing a trend towards significance (0.05<P<0.1) have been fitted with a sine wave by a least squares fitting method and the R^2 value is given; the permutation-based P value from JTK Cycle for significance of a day-night rhythm is given; significant data are shown in red and non-significant data are shown in black. The inset shows a schematic diagram of the α-adrenergic receptor pathway.

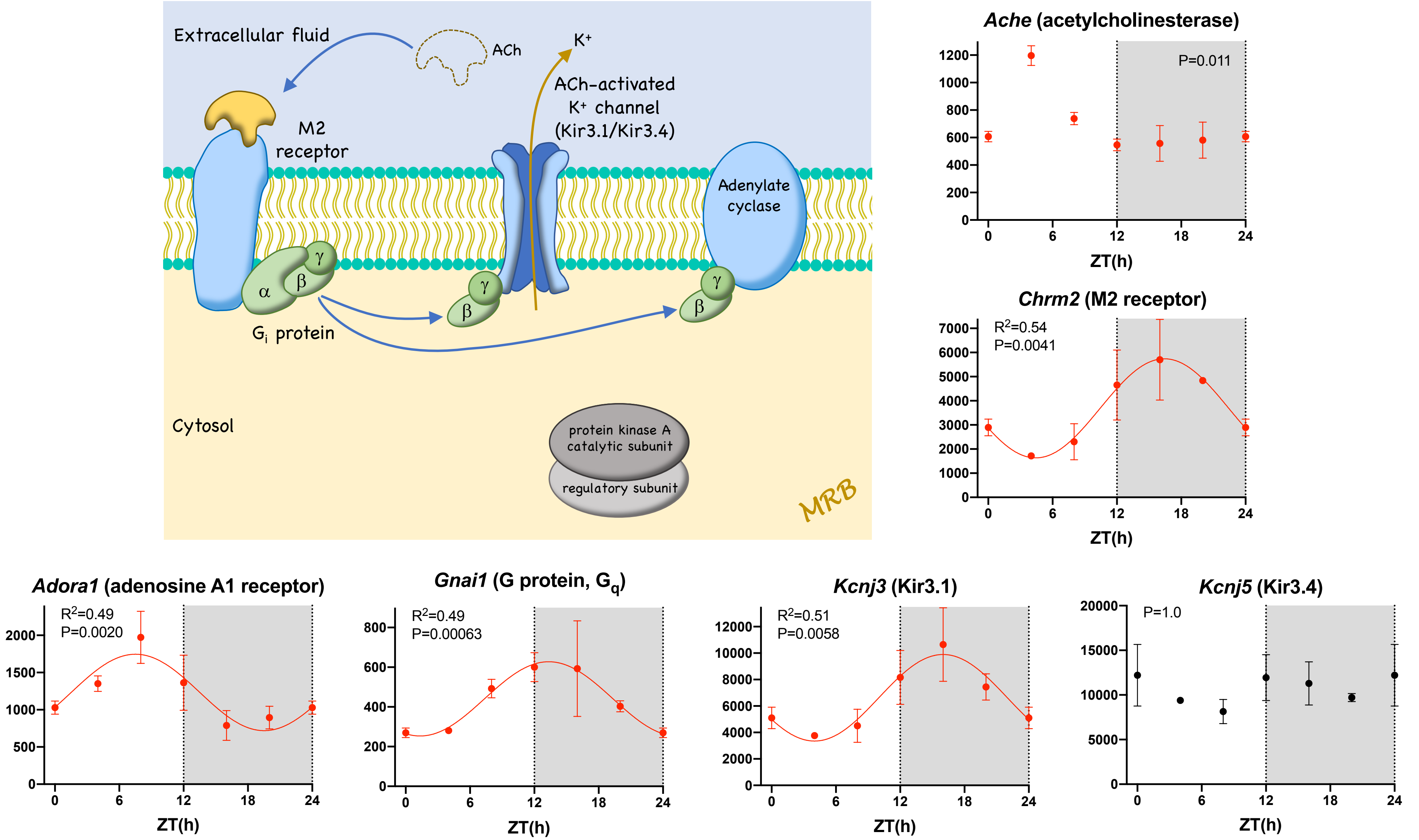

Fig. S6. Muscarinic receptor pathway. Abundance (normalised counts) of muscarinic receptor pathway transcripts is shown over 24 h. Gene name and common name (in parenthesis) given; mean ± SEM transcript abundance shown (n=3) at six ZT time points over 24 h (24 h data repeat of 0 h data); significant data (P<0.05) or data showing a trend towards significance (0.05<P<0.1) have been fitted with a sine wave by a least squares fitting method and the R^2 value is given; the permutation-based P value from JTK Cycle for significance of a day-night rhythm is given; significant data are shown in red and non-significant data are shown in black. The inset shows a schematic diagram of the muscarinic receptor pathway.

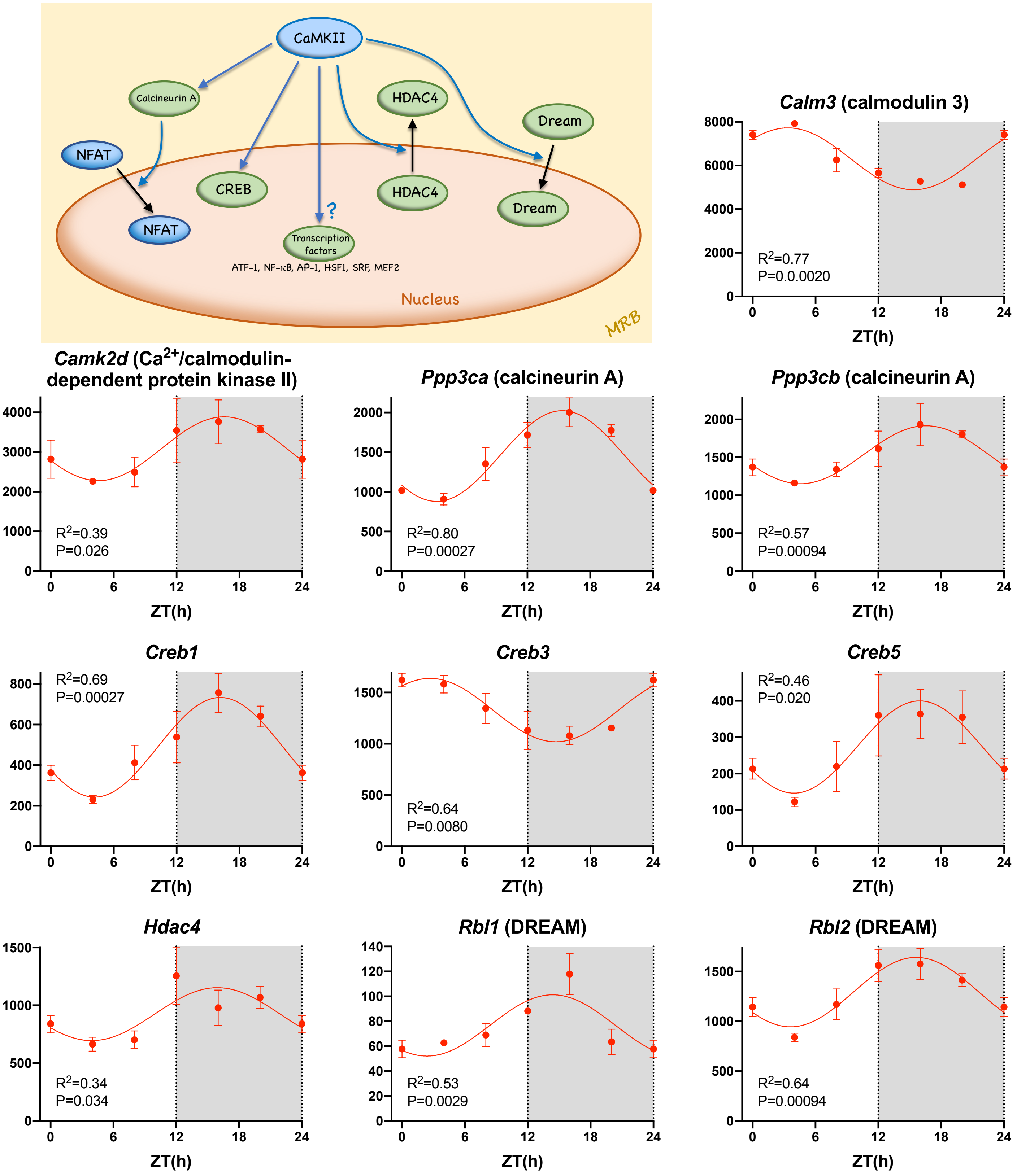

Fig. S7. CaMKII pathway. Abundance (normalised counts) of CaMKII pathway transcripts is shown over 24 h. Gene name and common name (in parenthesis) given; mean ± SEM transcript abundance shown (n=3) at six ZT time points over 24 h (24 h data repeat of 0 h data); all data are significant (P<0.05) and have therefore been fitted with a sine wave by a least squares fitting method and the R^2 value is given; the permutation-based P value from JTK Cycle for significance of a day-night rhythm is given; all data are significant and are therefore shown in red. The inset shows a schematic diagram of the CaMKII pathway (based on Kreusser and Backs[11]).

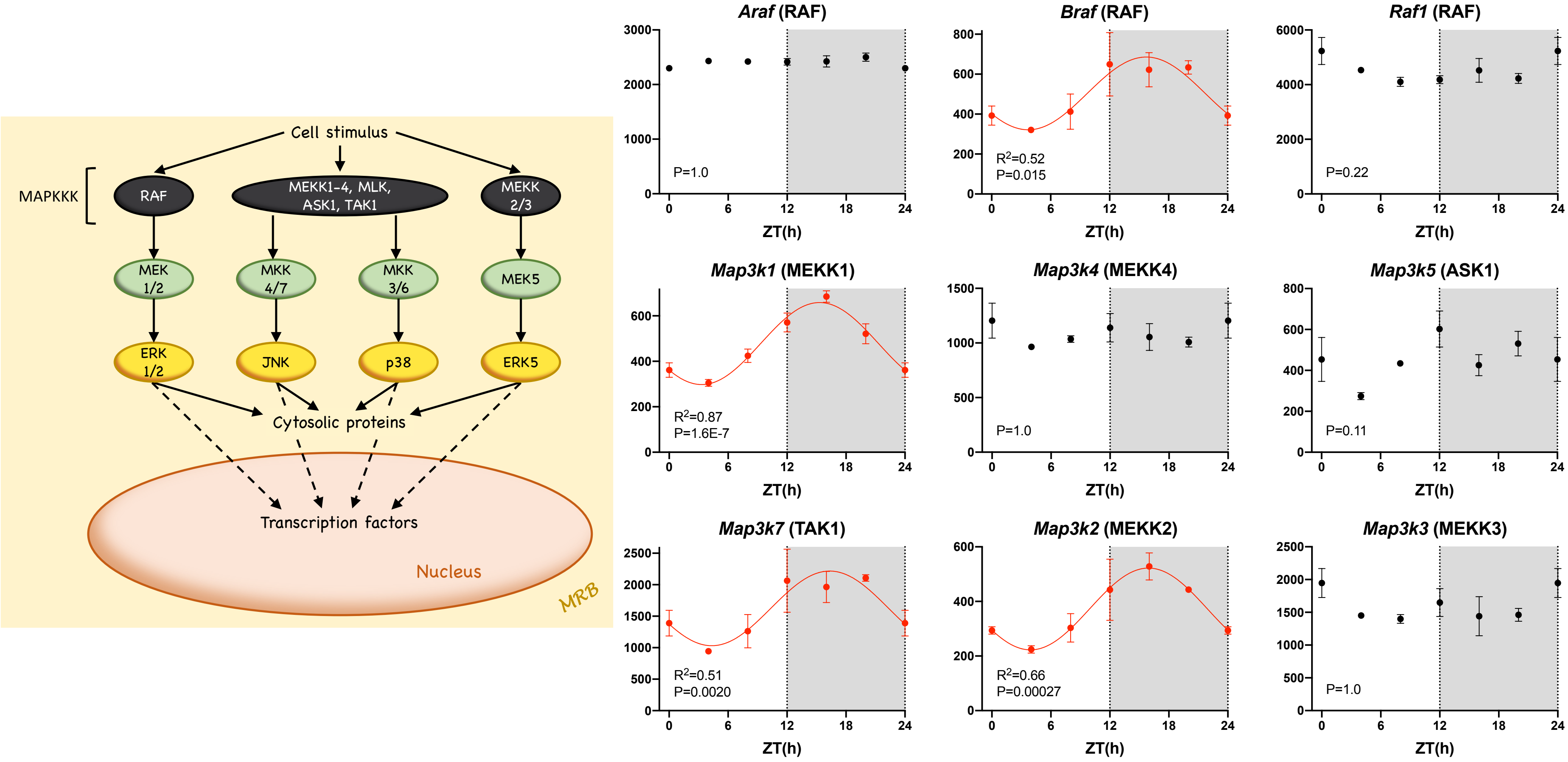

Fig. S8. First tier of kinases in the MAP kinase pathway. Abundance (normalised counts) of kinase transcripts is shown over 24 h. Gene name and common name (in parenthesis) given; mean ± SEM transcript abundance shown (n=3) at six ZT time points over 24 h (24 h data repeat of 0 h data); significant data ($P<0.05$) or data showing a trend towards significance ($0.05<P<0.1$) have been fitted with a sine wave by a least squares fitting method and the R^2 value is given; the permutation-based P value from JTK Cycle for significance of a day-night rhythm is given; significant data are shown in red and non-significant data are shown in black. The inset shows a schematic diagram of the MAP kinase pathway (based on Rose *et al.*[12]).

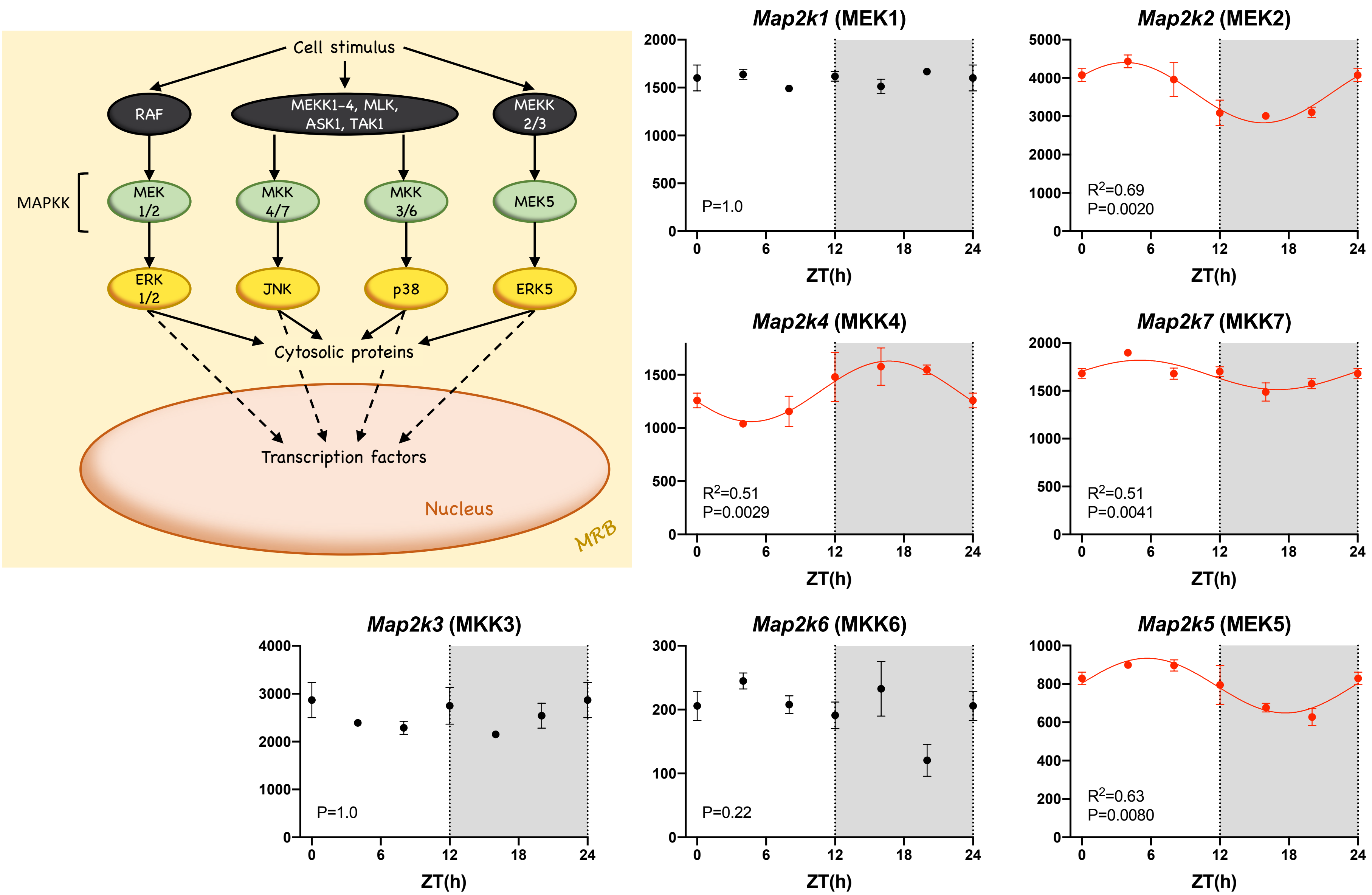

Fig. S9. Second tier of kinases in the MAP kinase pathway. Abundance (normalised counts) of kinase transcripts is shown over 24 h. Gene name and common name (in parenthesis) given; mean ± SEM transcript abundance shown (n=3) at six ZT time points over 24 h (24 h data repeat of 0 h data); significant data (P<0.05) or data showing a trend towards significance (0.05<P<0.1) have been fitted with a sine wave by a least squares fitting method and the R^2 value is given; the permutation-based P value from JTK Cycle for significance of a day-night rhythm is given; significant data are shown in red and non-significant data are shown in black. The inset shows a schematic diagram of the MAP kinase pathway (based on Rose *et al.*[12]).

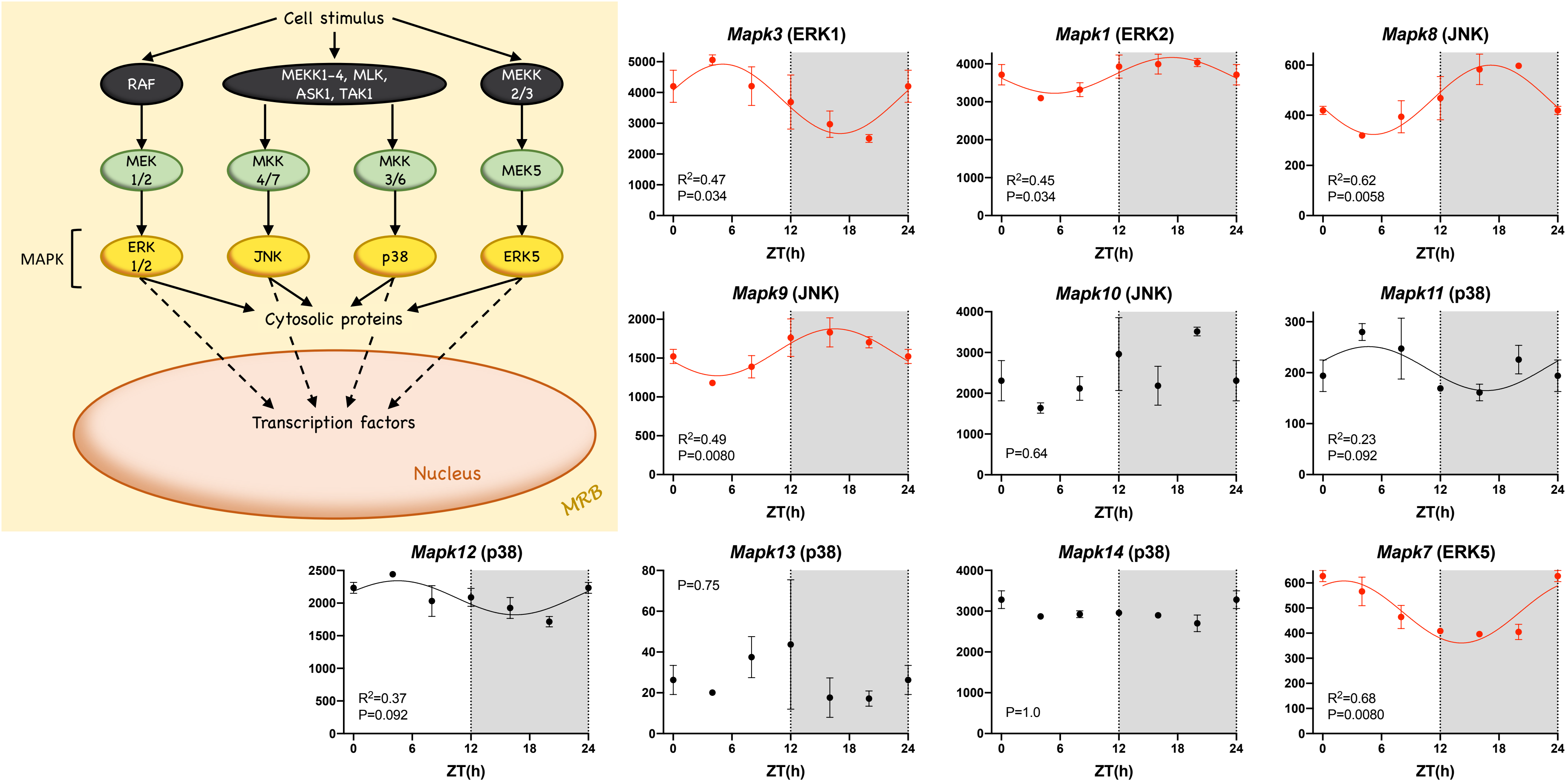

Fig. S10. Third tier of kinases in the MAP kinase pathway. Abundance (normalised counts) of kinase transcripts is shown over 24 h. Gene name and common name (in parenthesis) given; mean ± SEM transcript abundance shown (n=3) at six ZT time points over 24 h (24 h data repeat of 0 h data); significant data (P<0.05) or data showing a trend towards significance (0.05<P<0.1) have been fitted with a sine wave by a least squares fitting method and the R^2 value is given; the permutation-based P value from JTK Cycle for significance of a day-night rhythm is given; significant data are shown in red and non-significant data are shown in black. The inset shows a schematic diagram of the MAP kinase pathway (based on Rose *et al.*[12]).

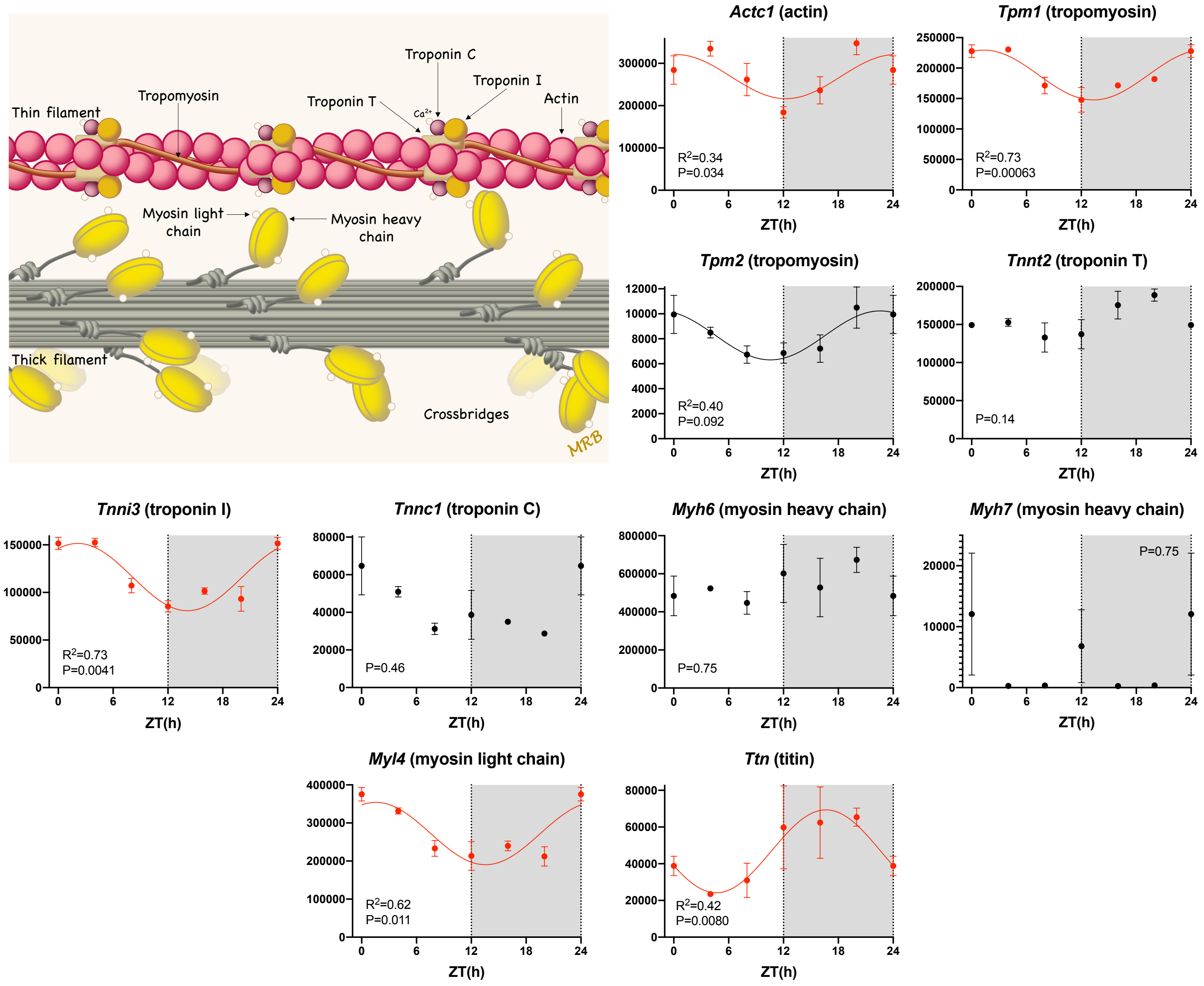

Fig. S11. Contractile proteins. Abundance (normalised counts) of transcripts for contractile proteins is shown over 24 h. Gene name and common name (in parenthesis) given; mean ± SEM transcript abundance shown (n=3) at six ZT time points over 24 h (24 h data repeat of 0 h data); significant data (P<0.05) or data showing a trend towards significance (0.05<P<0.1) have been fitted with a sine wave by a least squares fitting method and the R^2 value is given; the permutation-based P value from JTK Cycle for significance of a day-night rhythm is given; significant data are shown in red and non-significant data are shown in black. The inset shows a schematic diagram of the contractile apparatus (based on Redwood *et al.*[13]).

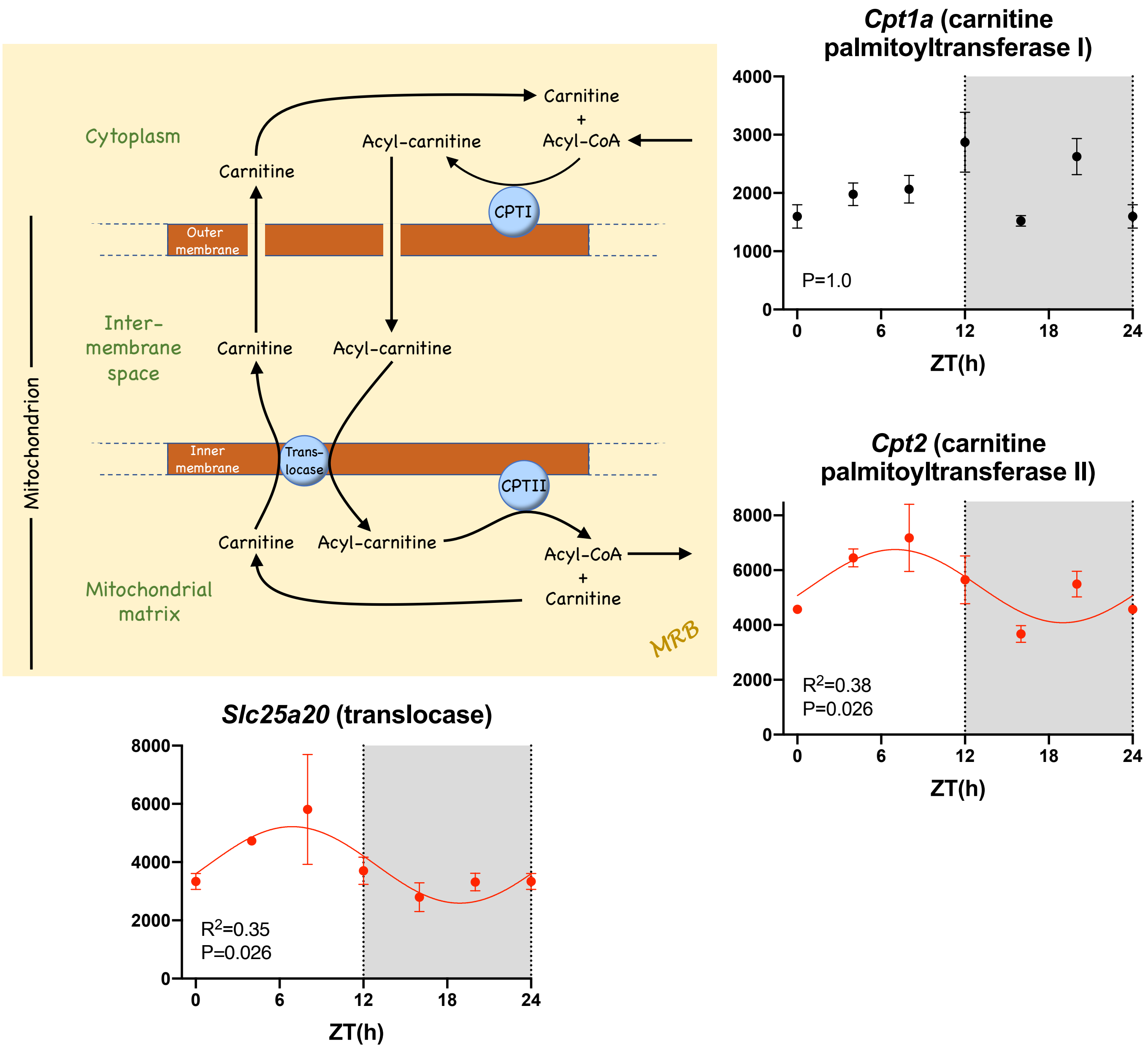

Fig. S12. Acyl-CoA transport from the cytoplasm to the mitochondrial matrix. Abundance (normalised counts) of acyl-CoA transport transcripts is shown over 24 h. Gene name and common name (in parenthesis) given; mean ± SEM transcript abundance shown (n=3) at six ZT time points over 24 h (24 h data repeat of 0 h data); significant data ($P<0.05$) or data showing a trend towards significance ($0.05<P<0.1$) have been fitted with a sine wave by a least squares fitting method and the R^2 value is given; the permutation-based P value from JTK Cycle for significance of a day-night rhythm is given; significant data are shown in red and non-significant data are shown in black. The inset shows a schematic diagram of acyl-CoA transport.

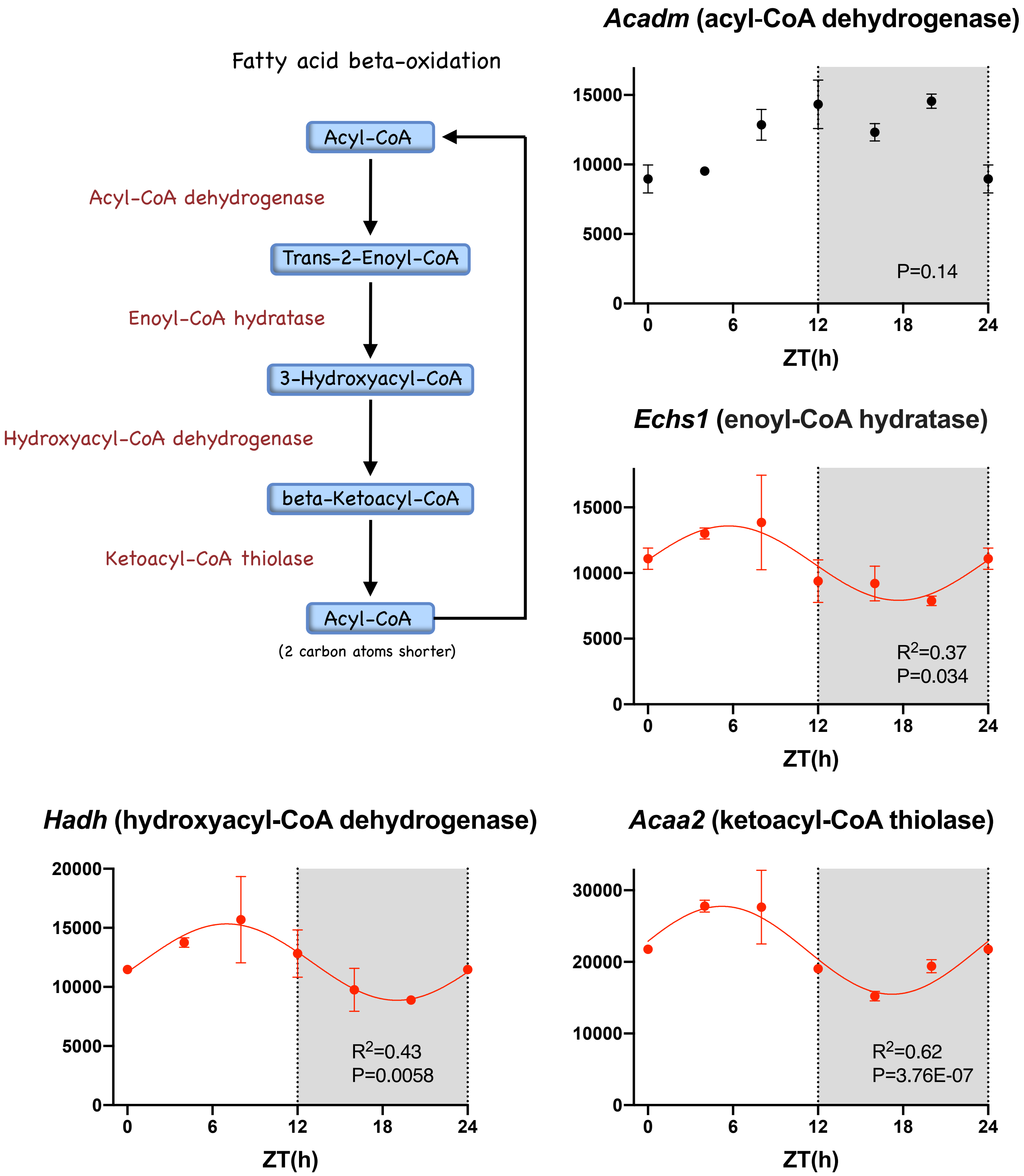

Fig. S13. Fatty acid β-oxidation. Abundance (normalised counts) of fatty acid β-oxidation transcripts is shown over 24 h. Gene name and common name (in parenthesis) given; mean ± SEM transcript abundance shown (n=3) at six ZT time points over 24 h (24 h data repeat of 0 h data); significant data (P<0.05) or data showing a trend towards significance (0.05<P<0.1) have been fitted with a sine wave by a least squares fitting method and the R^2 value is given; the permutation-based P value from JTK Cycle for significance of a day-night rhythm is given; significant data are shown in red and non-significant data are shown in black. The inset shows a schematic diagram of fatty acid β-oxidation.

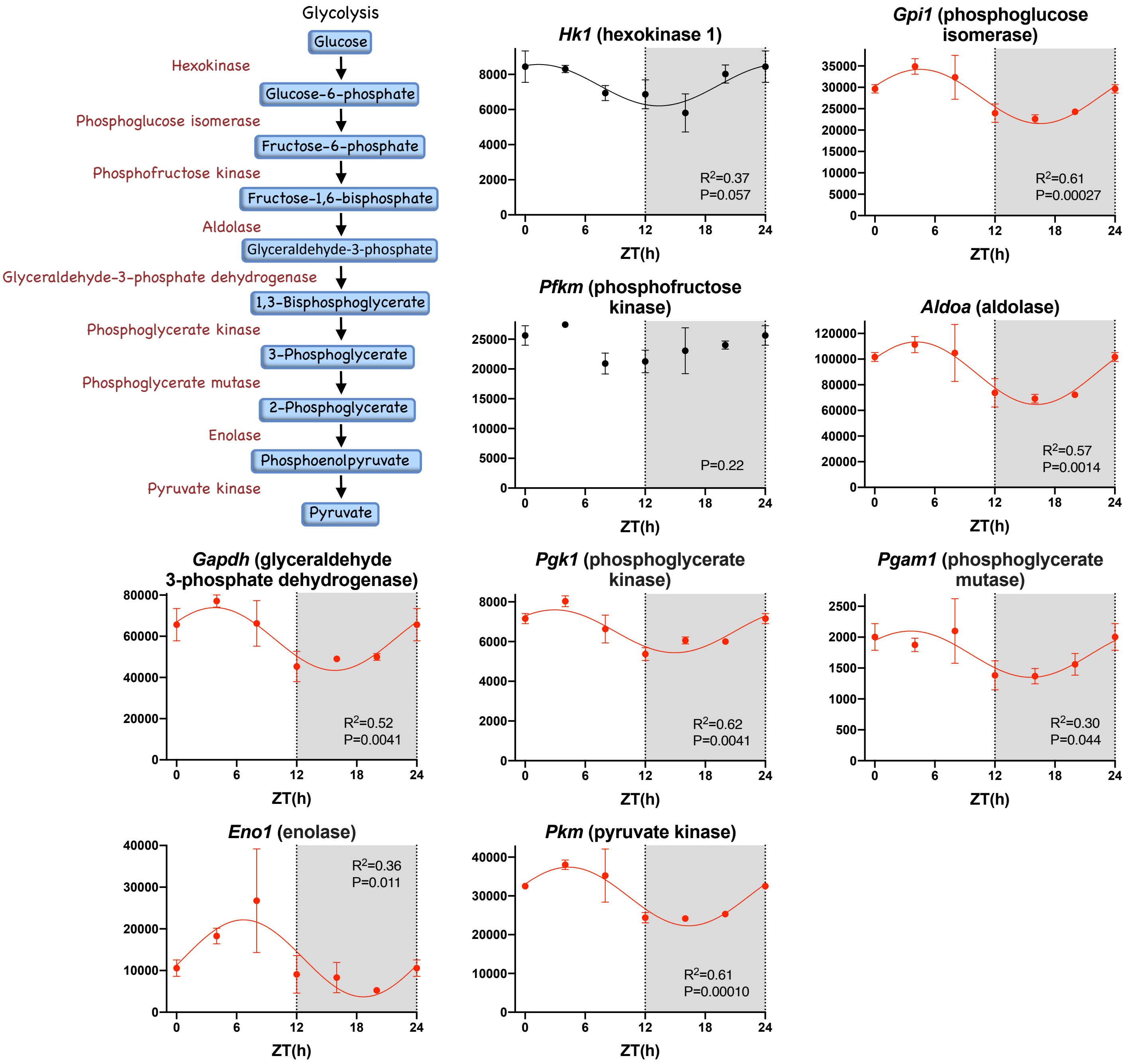

Fig. S14. Glycolysis. Abundance (normalised counts) of glycolysis transcripts is shown over 24 h. Gene name and common name (in parenthesis) given; mean ± SEM transcript abundance shown (n=3) at six ZT time points over 24 h (24 h data repeat of 0 h data); significant data (P<0.05) or data showing a trend towards significance (0.05<P<0.1) have been fitted with a sine wave by a least squares fitting method and the R^2 value is given; the permutation-based P value from JTK Cycle for significance of a day-night rhythm is given; significant data are shown in red and non-significant data are shown in black. The inset shows a schematic diagram of glycolysis.

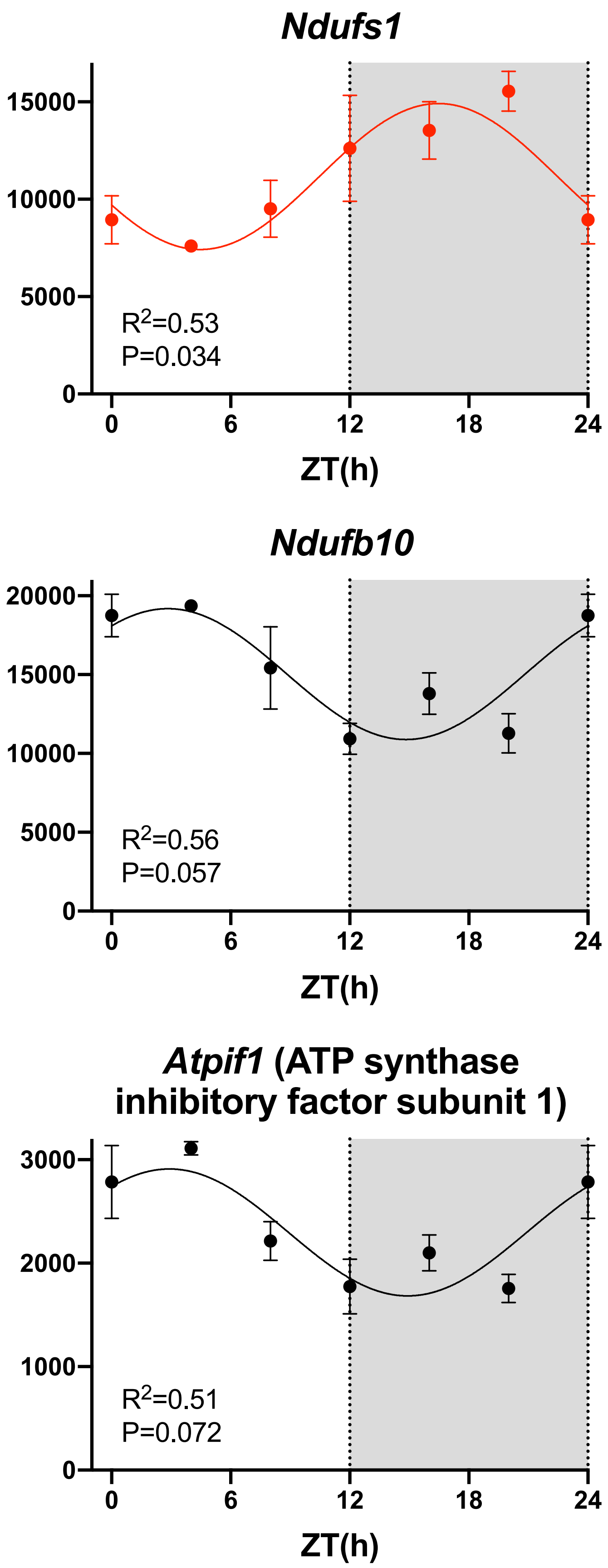

Fig. S15. Electron transport chain. Abundance (normalised counts) of two example electron transport chain transcripts and ATP synthase inhibitory factor subunit 1 is shown over 24 h. Gene name and common name (in parenthesis) given; mean ± SEM transcript abundance shown (n=3) at six ZT time points over 24 h (24 h data repeat of 0 h data); significant data ($P<0.05$) or data showing a trend towards significance ($0.05<P<0.1$) have been fitted with a sine wave by a least squares fitting method and the R^2 value is given; the permutation-based P value from JTK Cycle for significance of a day-night rhythm is given; significant data are shown in red and non-significant data are shown in black.

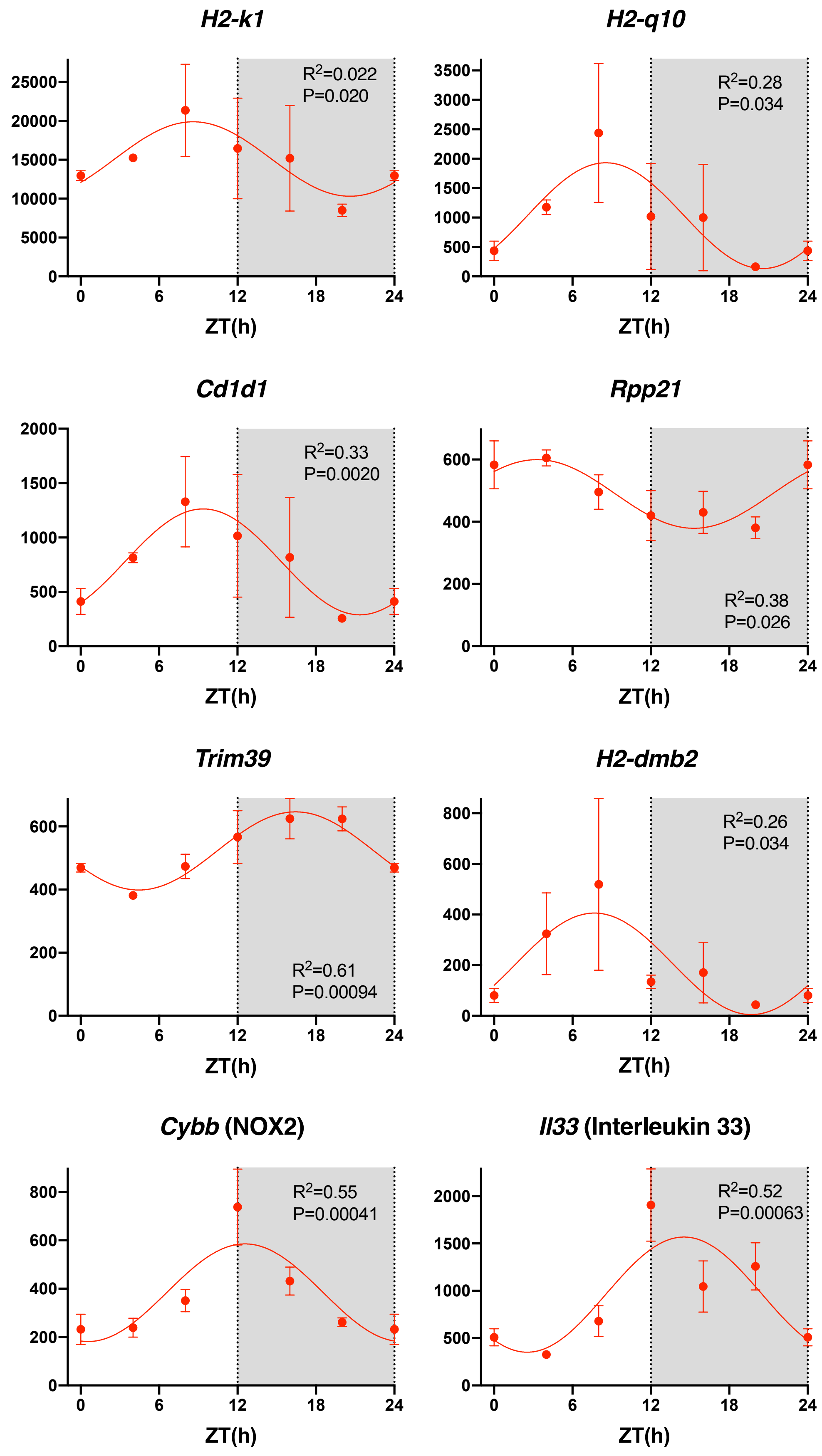

Fig. S16. Day-night rhythm in selected transcripts related to the immune system. Abundance (normalised counts) of transcripts shown over 24 h. Gene name and common name (in parenthesis) given; mean ± SEM transcript abundance shown (n=3) at six ZT time points over 24 h (24 h data repeat of 0 h data); all data are significant ($P<0.05$) and have therefore been fitted with a sine wave by a least squares fitting method and the R^2 value is given; the permutation-based P value from JTK Cycle for significance of a day-night rhythm is given; all data are significant and are therefore shown in red.

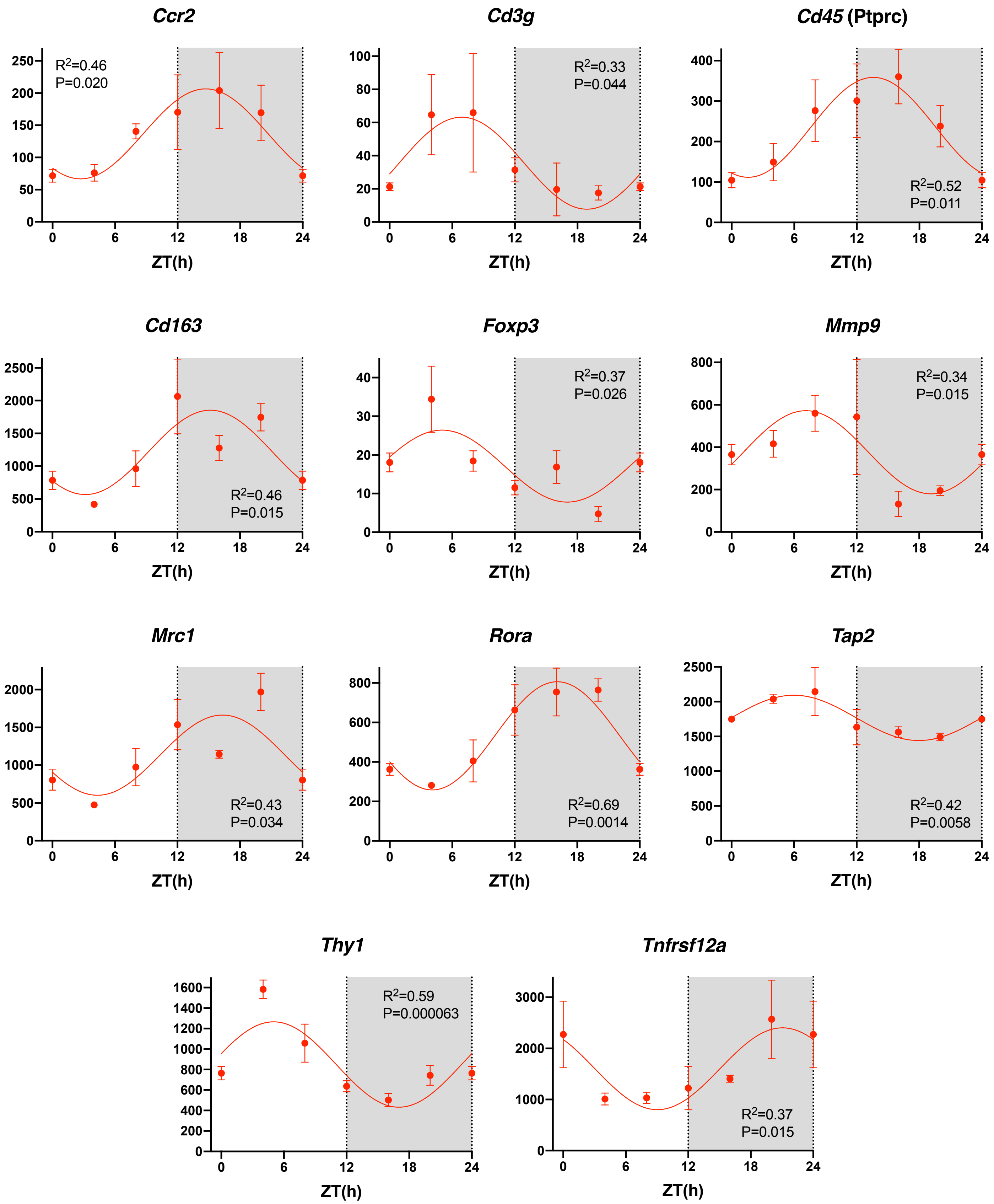

Fig. S17. Day-night rhythm in other transcripts related to the immune system. Abundance (normalised counts) of transcripts shown over 24 h. Gene name and common name (in parenthesis) given; mean ± SEM transcript abundance shown (n=3) at six ZT time points over 24 h (24 h data repeat of 0 h data); all data are significant ($P<0.05$) and have therefore been fitted with a sine wave by a least squares fitting method and the R^2 value is given; the permutation-based P value from JTK Cycle for significance of a day-night rhythm is given; all data are significant and are therefore shown in red.
